# A chromosome‐scale genome assembly reveals a highly dynamic effector repertoire of wheat powdery mildew

**DOI:** 10.1111/nph.15529

**Published:** 2018-11-14

**Authors:** Marion C. Müller, Coraline R. Praz, Alexandros G. Sotiropoulos, Fabrizio Menardo, Lukas Kunz, Seraina Schudel, Simone Oberhänsli, Manuel Poretti, Andreas Wehrli, Salim Bourras, Beat Keller, Thomas Wicker

**Affiliations:** ^1^ Department of Plant and Microbial Biology University of Zurich Zollikerstrasse 107 Zurich CH‐8008 Switzerland

**Keywords:** avirulence genes, *Blumeria graminis*, copy number variation, powdery mildew, recombination

## Abstract

- *Blumeria graminis* f. sp. *tritici* (*B.g. tritici*) is the causal agent of the wheat powdery mildew disease. The highly fragmented *B.g. tritici* genome available so far has prevented a systematic analysis of effector genes that are known to be involved in host adaptation. To study the diversity and evolution of effector genes we produced a chromosome‐scale assembly of the *B.g. tritici* genome.
- The genome assembly and annotation was achieved by combining long‐read sequencing with high‐density genetic mapping, bacterial artificial chromosome fingerprinting and transcriptomics.
- We found that the 166.6 Mb *B.g. tritici* genome encodes 844 candidate effector genes, over 40% more than previously reported. Candidate effector genes have characteristic local genomic organization such as gene clustering and enrichment for recombination‐active regions and certain transposable element families. A large group of 412 candidate effector genes shows high plasticity in terms of copy number variation in a global set of 36 isolates and of transcription levels.
- Our data suggest that copy number variation and transcriptional flexibility are the main drivers for adaptation in *B.g. tritici*. The high repeat content may play a role in providing a genomic environment that allows rapid evolution of effector genes with selection as the driving force.

## Introduction

*Blumeria graminis* is an ascomycete fungal pathogen that causes powdery mildew on cereals, one of the most important crop diseases. The species *B. graminis* encompasses at least eight *formae speciales* (ff. ssp.) that are defined by their specificity for different crops and wild grasses (Troch *et al*., 2012). Wheat powdery mildew *B. graminis* f. sp.* tritici* (*B.g. tritici*) and barley powdery mildew *B. graminis* f. sp. *hordei* (*B.g. hordei*) are economically the most important mildew forms. As obligate biotrophs, powdery mildews modify the host cells to feed on them while avoiding recognition by the host. For this fine‐tuned interaction, *B. graminis* relies on an arsenal of effector proteins. The delicate balance between maintaining virulence and avoiding host recognition is probably based on a rapid evolutionary turnover of effector genes through sequence diversification, duplications, deletions, and transcriptional regulation (Wicker *et al*., 2013; Menardo *et al*., 2017; Frantzeskakis *et al*., 2018). This high turnover supposedly contributes to the adaption of plant pathogens to the high selection pressure from rapidly changing agricultural environments.

Genome sequencing of multiple filamentous plant pathogens revealed that candidate effector genes are often located in distinct genomic compartments that have a high repeat content but are largely void of other genes (Dong *et al*., 2015; Möller & Stukenbrock, 2017). Based on these findings, the ‘two‐speed genome’ hypothesis was proposed, which states that the compartments rich in effector genes evolve at a faster pace than the rest of the genome (Dong *et al*., 2015; Wang *et al*., 2017). For example, in *Fusarium oxysporum*, effectors are preferably located on repeat‐rich accessory chromosomes (van Dam *et al*., 2017), whereas in *Leptosphaeria maculans* and *Phytophthora infestans* the effectors are found in repeat‐rich regions of the core chromosomes (Dong *et al*., 2015). It is assumed that mutation rates in effectors can be locally increased if they are near transposable elements (TEs) that are silenced through the repeat‐induced point mutation (RIP) pathway (Fudal *et al*., 2009). Such compartmentalization, however, seems not to be universally true: a recent study on the barley powdery mildew genome, based on new megabase‐scale sequence scaffolds, reported none of these characteristics, and the authors suggest that *B.g. hordei* has a ‘one‐speed’ genome (Frantzeskakis *et al*., 2018). Importantly, possible correlations of TEs and effector genes can only be studied in genomes of high contiguity.

Meiotic recombination has been proposed as an additional mechanism to accelerate gene evolution, as it is an important driver for genetic diversity in sexually reproducing organisms (Möller & Stukenbrock, 2017). Recombination occurs unevenly across the genome in most organisms; consequently, the effect of recombination on genetic diversity is uneven along the chromosome (Gaut *et al*., 2007; Mezard *et al*., 2015). Most data on meiotic recombination in fungi were obtained in studies of the budding yeast *Saccharomyces cerevisiae*, and the effect of recombination on genome evolution has only been addressed in a few phytopathogenic fungi (Croll *et al*., 2015; Van Kan *et al*., 2017; Laurent *et al*., 2018). However, a few studies have linked candidate effector evolution to recombination, since effectors tend to occur in recombination‐rich parts of the genome (Croll *et al*., 2015; Laurent *et al*., 2018).

Sequencing genomes of phytopathogenic fungi proved to be challenging due to their large size and high repeat content (Möller & Stukenbrock, 2017). Consequently, first assemblies of the barley and wheat powdery mildew genomes were highly fragmented (Spanu *et al*., 2010; Wicker *et al*., 2013). Although these genome assemblies allowed a detailed insight into the *B. graminis* gene content, only rudimentary analyses of the TE fraction and large‐scale chromosomal structures were possible. Recent advances in long‐read sequencing technology, along with new scaffolding methods, have enabled resolution of chromosome‐scale assemblies of an increasing number of plant pathogens’ genomes (Van Kan *et al*., 2017; Miller *et al*., 2018). Only genomes of high contiguity allow the addressing of topics such as gene space organization and copy number variation (CNV). These analyses are essential to be able to cover the entire diversity of the candidate effector complement of a pathogen. Such knowledge is important to allow one to determine gene redundancy in, for example, avirulence genes (S. Bourras *et al*., unpublished), as well as for diversity studies in the natural population of the pathogen.

Here, we present a chromosome‐scale assembly of the wheat powdery mildew genome that was produced by combining long‐read sequencing with high‐density genetic mapping and bacterial artificial chromosome data. This allowed detailed analyses of chromosomal macrostructures, distribution of recombination events, and effector gene family dynamics. We show that candidate effector genes of the same family occur in clusters and are enriched around recombination events. We identified a group of highly expressed candidate effector families with distinct characteristics, such as high levels of CNV in a global mildew population, sequence diversity, and transcriptional plasticity, suggesting involvement in host adaptation.

## Materials and Methods

### Fungal isolates and genetic mapping

Crossing of mildew isolates *B.g. tritici* 96224 (Switzerland) and *Blumeria graminis* f.sp. *triticale* isolate THUN‐12 (Poland) was done as previously described (Parlange *et al*., 2015). Ascospores from chasmothecia were ejected by exposure to wet Whatman paper for 1 wk at 20°C and 80% humidity. Single colonies from ascospores were collected on the susceptible wheat cv Kanzler and propagated by single‐colony isolation for two cycles to ensure clonal progeny. Progeny were grown on cv Kanzler at 4°C for long term storage. For comparison of genetic maps, KASP marker sequences (Bourras *et al*., 2015) were mapped to the Bgt_genome_v3.16 with blastn v.2.2.31+, (Camacho *et al*., 2009).

### Genome sequencing and variant calling

DNA was extracted from 10‐d‐old conidiospores using a cetyl trimethyl ammonium bromide/phenol–chloroform extraction (Bourras *et al*., 2015). Individual mapping population progeny were sequenced with an Illumina HISeq4000 at the Functional Genomics Center Zurich of the University of Zurich (FCGZ) to a *c*. 16‐fold coverage. Samples were pooled and indexed using Illumina TrueSeq Adapters. Raw reads were quality trimmed with sickle using default parameters (https://github.com/najoshi/sickle) and mapped against Bgt_genome_v3.16 using bowtie2 with parameters ‐L,‐0.6,‐0.25 (Langmead & Salzberg, 2012). The mapping files were sorted and duplicate reads removed using SAmtools view, sort and rmdup (Li *et al*., 2009). Read groups were added using picard tools (http://broadinstitute.github.io/picard). Owing to the low sequencing depth of some of the progeny, single nucleotide polymorphism (SNP) calling was done with freebayes using the parameters ‐p 1 ‐m 30 ‐q 20 ‐z 0.03 ‐F 0.7 ‐3 200 (Garrison & Marth, 2012). SNPs were subsequently filtered with vcftools (Danecek *et al*., 2011). SNPs with a quality score less than QC 40 and a minor allele frequency of < 0.2 were removed from the dataset. We allowed 10% missing data for each SNP. The vcf file was transformed into hapmap format using custom perl scripts (see also Supporting Information Methods S1).

High molecular weight DNA (12 μg) was used for library preparation and PacBio sequencing (performed at FCGZ). The assembly was done with falcon using a length cutoff for the initial mapping of 5000 and length cutoff for pre‐assembly of 7000 (Chin *et al*., 2016). Two Illumina runs of isolate 96224 were used to polish the PacBio assembly with pilon v.1.22 (Walker *et al*., 2014). Integrity of recombination hot spots in PacBio scaffolds was verified by PCR (Notes S1).

### Genome‐wide analyses

Guanine–cytosine (GC) content was calculated in 50 kb windows using the emboss program freak (http://www.bioinformatics.nl/cgi-bin/emboss/help/freak). Sequence coverage was calculated using the SAmtools depth command. Distances between genes were defined as the distance between the predicted start and stop codons of neighboring gene pairs. Genes located next to telomeres or centromeres were excluded from the two‐speed genome analysis.

We calculated recombination frequency in 50 kb windows as the maximum genetic distance between two markers in each window. For intervals without SNP markers, we estimated the recombination frequency based on the closest SNPs in flanking intervals. For details on genetic map construction, see Methods S1.

For repeat annotation we used pipelines that were previously described for barley (Wicker *et al*., 2017). TEs were classified according to Wicker *et al*. (2007). For the new TE families, consensus sequences were deduced using at least three and up to 100 complete copies. The centromeres of chromosomes 8 and 11 were manually annotated.

### *Blumeria g. tritici* gene diversity analyses

Genetic diversity of *B.g. tritici* genes was sampled in 36 previously published isolates (Table S3). Variant calling was done as described earlier. We used only SNPs in genes that were covered by at least five reads and where at least 95% of all reads contained the SNP in a given isolate. Ratios of nonsynonymous to synonymous changes were calculated according to Nei & Gojobori (1986). To estimate the mutation rate in genes, we only considered codons where the third base can be substituted freely by any base (Notes S2).

To estimate the number of duplicated genes, we performed blastn searches of gene sequences (including introns) against Bgt_genome_v3.16. All genes with multiple hits, full‐length coverage, and at least 100%, 98% or 95% identity, respectively, were retained.

Copy numbers of genes in individual isolates were estimated using genomic sequence coverage calculated for each gene (from start to stop codon) using SAmtools depth (with the ‐a option). Coverage per gene was normalized to the average coverage across all genes. A gene was considered deleted if coverage was < 0.1 and duplicated if coverage was > 1.7.

Additional methods on genetic map construction, transcriptome analysis, phylogenetic analysis, and statistics are available in Methods S1–S4.

### Data access

Bgt_genome_v3.16 is available at ENA (accession number: PRJEB28180). PacBio reads are available under the accession number PRJNA290428. Raw sequences of the progeny of the genetic map population are available under the accession number SRP148738.

## Results

### A combination of physical and genetic approaches yields a chromosome‐scale assembly of the *B.g. tritici* genome

**Figure 1:**
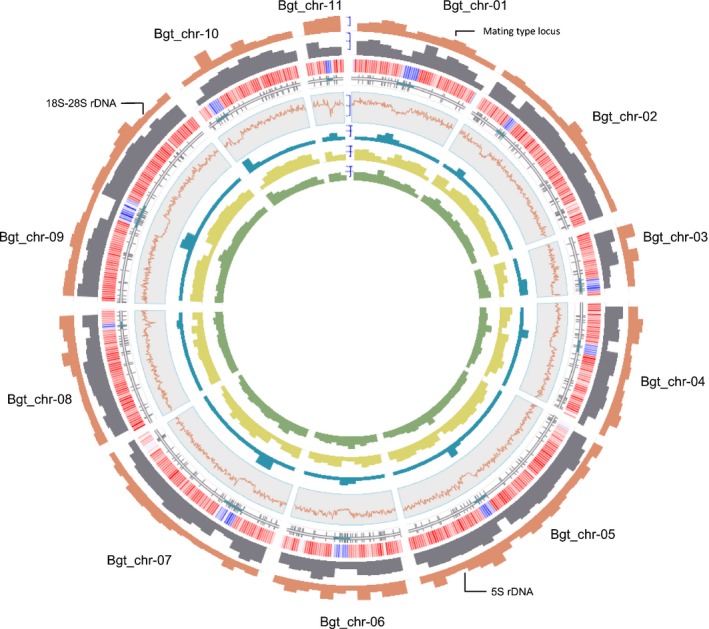
Overview of chromosomal features of the *Blumeria graminis* f. sp. *tritici* genome. Circular plot displaying the 11 chromosomes of *B.g*. *tritici* isolate 96224. The circular layers represent the following (from the outside to the inside): pink bar plot, total number of candidate effector genes in 1 Mb windows; gray bar plot, total number of genes in 1 Mb windows. The heatmap represents log‐transformed RNA sequencing coverage values from isolate 96224 in 200 kb windows (red, high coverage; blue, low coverage). Vertical gray lines indicate position of sequence gaps on the chromosomes. Blue regions mark the genetic centromeres. Red lines represent GC content estimated in 50 kb nonoverlapping windows. Blue bar plots represent long interspersed nuclear element (*LINE*) transposons of the family Fuji in kb/Mb in 1 Mb windows. Yellow bar plot represents total LINEs content in kb/Mb in 1 Mb windows. Green bar plots show total short interspersed nuclear element content in kb/Mb in 1 Mb windows.

Chromosome‐scale pseudomolecules of *B.g. tritici* isolate 96224 were assembled from 313 PacBio sequence contigs with the help of a genetic map (Table 1; see Notes S3 for assembly procedure). The genetic map is based on 123 159 SNPs and spans a total of 4910 cM in 11 linkage groups. The map was derived from a complete sequencing of 118 progeny of a cross between *B.g. tritici* isolates 96224 and *B.g. triticale* isolate THUN‐12 (Methods S1). THUN‐12 was chosen for the cross because of its high level of dissimilarity with 96224 (Menardo *et al*., 2016). The scaffolding of the pseudomolecules was complemented with bacterial artificial chromosome end sequencing and fingerprinting information (Wicker *et al*., 2013). The 313 contigs had an N50 of 873 kb (N90 of 223.7 kb) and represent 97.6% of the total sequence (Table 1). The 11 pseudomolecules (hereafter equated with chromosomes) range in size from 3.6 to 19.7 Mb (Fig 1; Table S1) and contain only 357 sequence gaps. The final genome version is hereafter referred to as Bgt_genome_v3.16.

**Table 1:** Statistics of the *Blumeria graminis* f. sp. *tritici* genome assembly

	PacBio raw assembly	Scaffolds anchored in chromosomes	Unanchored scaffolds
Total scaffolds	653	313	340
Total length^a^	140 575 254	137 242 670	3332 584
Largest scaffold^a^	4473 759	4473 759	93 937
Average size^a^	215 276	438 474	9801
N50^a^	847 964	873 016	13 852
N90^a^	190 430	223 677	4522
Percentage of the sequences^b^		97.63%	2.37%

a Scaffold sizes are indicated in bp.

b Indicates the percentage of the raw assembly that is either anchored or unanchored.

The genome assembly has a size of 140.6 Mb. However, we estimate the actual genome size to be *c*. 166.6 Mb, because the assembly contains *c*. 2.2 Mb of collapsed tandem repeats that represent an estimated 25.8 Mb of sequence (Table S2; Notes S4). To test for the possibility of accessory chromosomes, we mapped genomic Illumina reads of 35 publicly available *B.g. tritici*, 22 *B.g. triticale*, and five *Blumeri graminis* f.sp. *secalis* isolates (Menardo *et al*., 2016; Praz *et al*., 2017) (Tables S3, S4) onto the assembly. We observed an overall even sequence coverage for all chromosomes in all isolates, suggesting that none of the 11 chromosomes is missing in any isolate. Furthermore, chromosome size strongly correlates with gene content, showing that there are no repeat‐rich/gene‐poor chromosomes (Fig. S1a). Together, these data suggest that none of the 11 chromosomes is a dispensable accessory chromosome.

**Figure 2:**
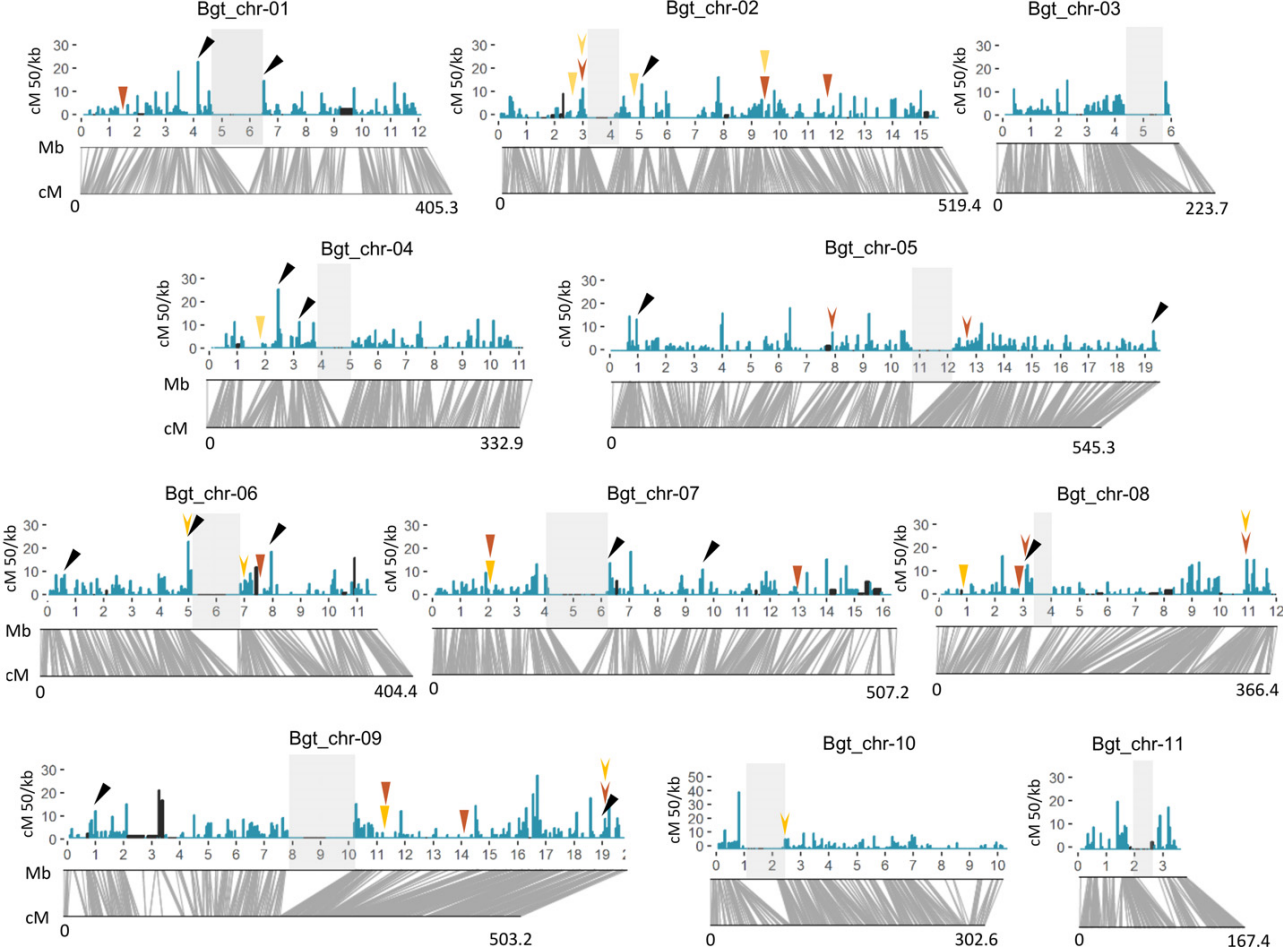
Genome‐wide distribution of recombination frequencies in the 96224 × THUN‐12 population. Recombination frequencies for the cross 96224 × THUN‐12 were calculated in 50 kb nonoverlapping windows and depicted in blue bars. Intervals depicted in black represent physical intervals for which no single nucleotide polymorphism marker was available and recombination frequencies were estimated based on the flanking intervals. Underneath, the collinearity between physical in megabase pairs (Mb) and the genetic distance in centimorgans (cM) of the chromosomes is depicted in gray lines. The genetic centromere is depicted by a gray rectangle for each chromosome. Black arrowheads indicate the position of the PCR amplified regions (see Supporting Information Notes S1). Colored arrowheads represent regions with conserved recombination frequencies between the 96224 × THUN‐12 mapping population with two earlier mapping populations. Red represents regions with conserved recombination frequencies between 96224 × THUN‐12 and 96224 × JIW2. Yellow represents regions with conserved recombination frequencies between 96224 × THUN‐12 and 96224 × 94202. Arrowheads represent regions in which the recombination frequencies in 96224 × 94202 or 96224 × JIW are higher than 3 cM/50 kb and also high in 96224 × THUN‐12. Dented arrowheads represent regions in which recombination frequency is equal to 0 cM/50kb in 96224 × 94202 or 96224 × JIW and 96224 × THUN‐12.

Approx. 85% of the *B.g. tritici* genome is derived from TE sequences (Notes S5; Fig. S2). Different TE superfamilies and families are evenly distributed along chromosome arms and interspersed with genes. By contrast, centromeres could be well defined: they are nonrecombining regions ranging in size from 683 kb to 2.4 Mb (Figs 1, 2) that have a lower GC content than the rest of the chromosome (Fig. S3). Furthermore, they are practically void of genes and highly enriched in 11 families of non‐long‐terminal‐repeat retrotransposons (Fig. 1). These centromeric TEs are interspersed with two types of centromeric tandem repeats*: CentA* has a size of 189 bp and is found on Chr‐01 and Chr‐08, whereas *CentB* has a size of 197 bp and is found on Chr‐09 and Chr‐11 (Notes S4; Figs S4, S5; Table S5). The two different types of centromeric tandem repeats could be remnants of an ancient hybridization event that was postulated previously (Menardo *et al*., 2017; Notes S4). At the ends of seven chromosomes, we found 300–400 bp of the canonical TAACCC telomeric repeats, indicating that PacBio technology can successfully cover some chromosome ends.

### *Blumeria g. tritici* contains a higher number of genes than previously estimated

A complete reannotation of the *B.g. tritici* genome identified 8470 genes, *c*. 30% more than previously reported (Praz *et al*., 2017). The genome assembly covers 98% of the core ascomycete genes, comparable to the high‐quality assemblies of *B.g. hordei* (Frantzeskakis *et al*., 2018; Fig. S6). We particularly focused on the reannotation of candidate effector gene families using the approach previously described (Praz *et al*., 2017; Notes S6), which assumes that candidate effector proteins contain a signal peptide and evolved in a specific host–pathogen interaction. Thus, they are expected to have little or no homology to proteins outside the species. We clustered the *B.g. tritici* proteins with proteins from barley powdery mildew (*B.g. hordei*), *Neurospora crassa* and *Podospora anserina*, the latter two being two nonpathogenic ascomycetes for which we do not expect to find homologues to *B.g. tritici* genes. We identified 235 candidate effector families containing only genes from *B.g. tritici* (1304 genes) or *B.g. hordei* (667 genes), but no homologues in *N. crassa* and *P. anserina*, of which at least one family member has a signal peptide. The gene models of these 1304 effector genes were manually curated. A total of 460 genes were classified as ‘weak’ effectors (WEs) due to low prevalence of signal peptide in the family (< 10%) or homology to TEs. These 460 genes were excluded from subsequent analysis of candidate effectors. Thus, the final complement of candidate effectors in *B.g. tritici* consists of 844 genes, out of a total of 8470 predicted genes. Only 3.7–5.1% of the candidate effector genes have homologues in *Botrytis cinerea* and *Phialocephala subalpina*, despite both of them being Leotiomycetes (the same taxonomic group as *B.g. tritici*). This suggests that candidate effector genes are indeed largely species specific (Notes S6; Table S6).

In addition to protein‐coding genes, Bgt_genome_v3.16 provides good coverage of genes for the ribosomal backbone (rDNA): a cluster of 45S rDNA genes is located on Chr‐09 (Fig. S5) and a cluster of 5S rDNA is found on Chr‐05. The high coverage of these regions indicates that they contain collapsed reads and that *B.g. tritici* encodes for *c*. 800 copies of 45S and 1300 copies of the 5S ribosomal cluster (details in Notes S4). This is a high number compared with yeast, which has only *c*. 150 copies of the 45S cluster in its genome (Kobayashi *et al*., 1998).

### Recombination hot spots are conserved between three mapping populations

We used the genetic map of the mapping population 96224 × THUN‐12 to study the distribution of recombination in *B. graminis* (see Methods S1). Recombination rate per chromosome varied between 28.07 cM/Mb for Chr‐05 and 46.02 cM/Mb for the smallest chromosome Chr‐11 (Table S7). Overall, smaller chromosomes displayed a higher recombination rate than larger chromosomes (*R* ^2^ = 0.6731, Fig. S7a,b).

We also observed high variation of recombination rates along the chromosomes. We estimated recombination rates in 50 kb windows along the 11 chromosomes (Fig. 2) and we identified 72 recombination hot spots (defined as regions with > 10 cM/50 kb) and 1642 windows in which no recombination occurred. A total of 300 of these nonrecombining windows corresponded to centromeric regions, which tend to be larger in bigger chromosomes (*R* = 0.3192, Fig. S7c). The remaining 1342 nonrecombining 50 kb windows, corresponding to 85.2 Mb of sequence, are outside the centromeres and will hereafter be referred to as recombination cold spots. One cold spot on Chr‐01 (between positions 6671 731 and 6897 291) contains the mating‐type locus (Fig. 1). Thus, recombination occurred in < 40% of the genome in the 96224 × THUN‐12 mapping population. Of the 72 identified recombination hot spots, 17 were found within 500 kb of a centromere, 7 within 500 kb of a chromosome end, and 48 in between (Fig. 2). The proportion of recombination hot spots flanking the centromeres was significantly higher than expected if hot spots were randomly distributed (under random distribution, 5.7 hot spots would be found flanking centromeres, *χ* ^2^ goodness‐of‐fit test, *P* = 0.000009). Indeed, all except chromosome 9 contain at least one hot spot near the centromeres. The genome assembly in 19 hot spots was verified by PCR to exclude assembly artefacts (see Notes S1; Figs 2, S8; Tables S8, S9).

The newly emerged f.  sp. *B.g. triticale* resulted from a hybridization between *B.g. tritici* and *B.g. secalis* (Menardo *et al*., 2016). Consequently, its genome consists of a mosaic of alternating fragments originating from either parental ff. ssp. Sequences of distantly related species can be impaired in their ability to recombine (Koo *et al*., 2017). However, we found that recombination is only slightly lower in regions in which THUN‐12 consists of the genomic makeup of *B.g. secalis* compared with *B.g. tritici* (1.5 cM/50 kb and 1 cM/50 kb, respectively) (Table S10). Thus, we concluded that chromosomes of *B.g. tritici* and *B.g. secalis* are able to freely recombine. Interestingly, the transitions from *B.g. secalis* and *B.g. tritici* genotypes in THUN‐12 are located in regions of high recombination rate (4.55 cM/50 kb), suggesting that high recombination regions are conserved between the initial parental cross of the hybrid *B.g. triticale* and the 96224 × THUN‐12 population.

**Figure 3:**
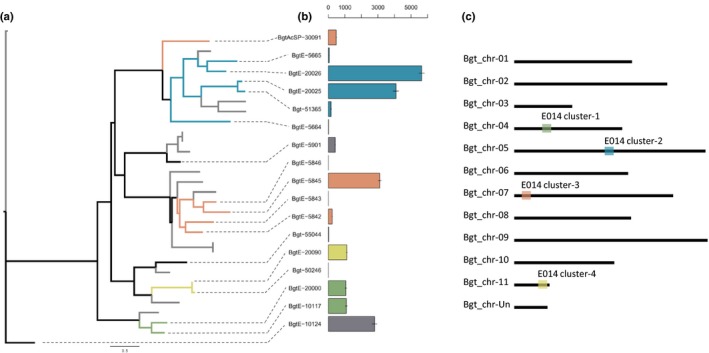
Effector family E014 is organized in four clusters. (a) Phylogenetic tree of candidate effector family E014. Gray branches correspond to *Blumeria graminis* f. sp. *hordei* genes. *Blumeria graminis* f. sp. *tritici* genes are depicted with either black branches (single genes) or with colored branches that correspond to different genomic clusters. (b) Gene expression of each *B.g*. *tritici* family member in reads per kilobase pairs per million reads based on three biological replicates. The error bars represent ± SE of the mean. Gene expression varies drastically between family members as well as between genes of the same cluster. (c) Genomic organization of the different clusters of candidate effector family E014. The color boxes correspond to the different clusters depicted in the phylogenetic tree in (a).

To further test if recombination hot spots and cold spots are conserved in *B. graminis*, we compared the 96224 × THUN‐12 genetic map with two earlier genetic maps sharing the parental isolate 96224 (96224 × 94202 and 96224 × JIW2, published in Bourras *et al*., 2015). These two maps are based on smaller numbers of markers, and therefore only a limited number of regions were comparable. Nevertheless, we identified 13 regions (seven in 96224 × 94202 and six in 96224 × JIW2) in which recombination rate was high (> 3 cM/50 kb). All but one of the corresponding physical regions in 96224 × THUN‐12 also exhibit high recombination frequencies (Fig. 2; Table S11). Interestingly, two of these high‐recombining regions are conserved between the three populations. We also identified 30 recombination cold spots in the 96224 × 94202 and 96224 × JIW2 mapping populations. For these 30 recombination cold spots, the majority of the corresponding regions in 96224 × THUN‐12 also exhibited low recombination rate (Fig. 3; Table S12). Both high recombining regions and cold spots are significantly more conserved between the mapping populations than they would be if these regions were randomly distributed (*χ* ^2^ goodness‐of‐fit test *P* < 0.05; Tables S11, S12; Methods S4). Thus, the genomic regions where recombination events occur seem to be conserved between populations that share one parental isolate. This suggests the existence of a mechanism that controls the physical location of recombination in *B. graminis*.

### Candidate effector clusters display a wide range of gene expression levels

On a genome‐wide scale, candidate effector genes were uniformly distributed and their number per chromosome correlated well with chromosome length (Fig. S1). However, members of the same candidate effector gene families are often found in gene clusters (defined as at least two genes of the same family that are separated by < 100 kb; Figs 3, S9, S10). Indeed, for the 16 largest effector families, we identified at least one physical cluster. For instance, family E014 consists of 13 members in four different clusters and an additional four ‘solitary’ genes (Fig. 3). The clusters are distributed on four different chromosomes and contain five genes (on Chr‐05), four genes (on Chr‐07) and two genes (on Chr‐04 and Chr‐11) (Fig. 3). In the candidate effector gene family E003 (68 genes), a cluster consisting of 27 genes covers an entire arm of Chr‐11 (Fig. S9). To test if clustered family members are phylogenetically more closely related to each other than to other family members, we inferred phylogenetic trees for the 16 largest candidate effector gene families (examples in Figs 3, S10). Correlation between genetic and physical distance of these 16 families was highly significant except for families that consist of only one cluster (Mantel test *P*‐value < 0.05, Table S13; see Methods S4). These data indicate that candidate effector gene clusters in *B.g. tritici* arise from local gene duplications.

To test if family members within clusters are co‐regulated, we analyzed expression patterns within and between candidate effector families. Here, we used published transcriptome data from isolate 96224 growing on the susceptible wheat cv Chinese Spring (Praz *et al*., 2018) at 48 h post infection (when the haustorium is formed and the host–pathogen interaction is established). We observed a wide range of expression levels within individual families (Figs S3, S11). All families include groups of highly and lowly expressed genes. Interestingly, highly expressed genes co‐occur with genes expressed at low levels or inactive genes in physical clusters. For instance, in family E014, the largest cluster on Chr‐05 contains two highly expressed genes and three members that are expressed at low levels (Fig. 3). Thus, expression is not regulated at the cluster level but is highly variable between individual genes both at family level and the cluster level.

### Candidate effector gene clusters undergo expansion

**Figure 4:**
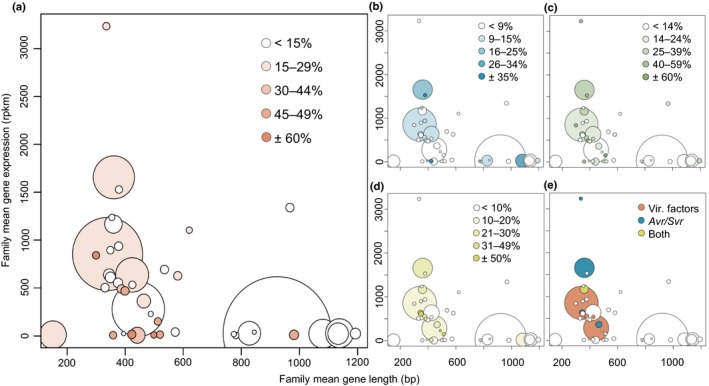
Transcriptional and copy number variation plasticity in candidate effector gene families in *Blumeria graminis* f. sp. *tritici*. Relationship of gene length and expression levels in candidate effector gene families in *B.g*. *tritici*. The size of the bubbles indicates the number of genes in the candidate effector gene families. The *x*‐axis represents the mean gene length in each effector family in base pairs and the *y*‐axis represents the mean gene expression in each candidate effector family based on three RNA sequencing biological replicates in reads per kilobase pairs per million reads (rpkm) of the reference isolate 96224. (a) The coloring represents the proportions of family members that are duplicated and share at least 98% identity to another gene in the reference genome. (b) The coloring represents the proportions of family members that are deleted in other *B.g*. *tritici* isolates compared with the isolate 96224. (c) The coloring represents the proportions of family members that are duplicated in other *B.g*. *tritici* isolates compared with the isolates 96224. (d) The coloring represents the proportions of family members that are differentially expressed among three different *B.g. tritici* isolates. (e) The families containing known avirulence genes or suppressor of avirulence genes in *B.g. tritici* or homologues of such genes in *Blumeria graminis* f. sp*. hordei* are colored in blue. The families containing homologues of known virulence factors in *B.g. hordei* are colored in pink. The yellow color represents family E014 that contains two *Avrs* and two virulence factors.

Despite the high variation in expression levels within candidate effector families, families of effector genes could be classified in two different groups based on the average gene length per family (Fig. 4). Interestingly, gene family lengths in these groups also correlate with mean expression levels of the families (Fig. 4, Fig. S12). Group 1 contains short (100–200 aa) and highly expressed proteins (mostly > 250 reads per kilobase of transcript per million mapped reads, rpkm) and group 2 contains larger (> 300 aa) and less expressed (< 250 rpkm) proteins. Out of the 16 largest families, only family E016 could not be assigned to one of the two groups. Family E016 contains very short and low expressed genes. Interestingly, all cloned avirulence genes (*Avrs*) in *B.g. tritici* (four) and homologues of *B.g. hordei Avrs* (two), as well as the suppressor of avirulence *SvrPm3*, are group 1 genes (Fig. 4e) (Bourras *et al*., 2015, unpublished; Lu *et al*., 2016; Praz *et al*., 2017). In addition, homologues of seven effector genes, for which virulence function was experimentally shown in *B.g. hordei*, belong to group 1 (Pliego *et al*., 2013; Ahmed *et al*., 2016; Pennington *et al*., 2016).

Candidate effector genes display higher rates of deletions and duplications than noneffector genes do (Menardo *et al*., 2017). To quantify turnover rates in group 1 and group 2 effectors, we identified gene duplications in the reference assembly, considering genes as duplicated if they appear in at least two identical copies (100% identity including introns) in the 96224 assembly. We identified 290 genes that are present in more than one identical copy (Table S14). Among these, group 1 candidate effectors (28 of 290, exact binomial test *P* = 0.0006) but not group 2 (three of 290, *P* = 0.074) candidate effectors are significantly enriched (exact binomial test *P* < 0.05). If gene duplications occur locally, they contribute to gene cluster expansion. Indeed, upon manual inspection of the 31 duplicated genes of group 1 and 2 candidate effectors, we discovered that five duplications occurred in tandem, and for six duplications the two genes are located next to each other but in inverted orientation. Notably, we found three cases in which the candidate effectors were duplicated to another chromosome. To test for recent but already diverged gene duplications, we identified gene pairs that share at least 98% sequence identity (Table S15). Among 714 such pairs, we found again that group 1 candidate effectors are strongly enriched (Table S15; Fig. 4a). Thus, our data suggest that recent expansion of gene clusters predominantly occurs in group 1 families.

### Candidate effector genes show high levels of copy number polymorphisms in a world‐wide population

Because of the evidence for frequent and recent gene duplications already described, we studied CNV in a population of 36 previously sequenced *B.g. tritici* isolates originated from Switzerland (three isolates, including 96224), the UK (one isolate), Israel (10 isolates), and China (22 isolates) (Table S3). Using sequence coverage as estimate for CNV (see the Materials and Methods section), we estimated that 3.5% of the genes are deleted and 10.2% are duplicated in at least one isolate of *B.g. tritici* (Table S15). As control, we used six housekeeping genes (Pennington *et al*., 2016) for which no CNV is expected (Fig. S13). The rate at which CNV occurs varied between different gene classes. For instance, in candidate effector family 008, the family of the *AvrPm3a2/f2*, about one‐third of the genes are duplicated in at least one isolate. Both duplications and deletions of genes are strongly enriched in group 1 effectors (exact binomial test *P* < 0.05) (Fig. 4; Table S15). Approx. 37% of the gene duplications are specific to one single isolate and half of the duplications occur in less than half of the isolates, suggesting a high rate of isolate‐specific gene expansion (example in Fig. S14). Furthermore, candidate effector genes have higher ratios of nonsynonymous to synonymous substitutions than noneffector genes do, but they show no overall higher nucleotide substitution rate than noneffector genes (Notes S2; Tables S16–S18; Fig. S15).

In addition to genomic variations, we also wanted to study expression levels, because Bourras *et al*. (2018) proposed that expression differences represent an additional level of plasticity in candidate effector genes. We therefore analyzed differential gene expression between three *B.g. tritici* isolates (96224, 94202, JIW2) on cv Chinese Spring, published in Praz *et al*. (2018) at the time of haustorium formation (2 d after infection). Among the 339 differentially expressed genes between two isolates (|log FC| > 1.5 and *P*‐value < 0.01), 87 are effector genes (25.7%, enrichment). Interestingly, 65 of the 87 differentially expressed effectors are from group 1 and only 16 are from group 2, a significant enrichment (binomial test, *P* < 0.05; Fig. 4; Table S19). Gene duplications can influence the measure of gene expression since transcripts from identical gene copies are assorted to the same gene in the reference assembly. In our analysis, only 6.9% of the differentially expressed genes also carry a CNV, suggesting that the majority of the genes we identified are real differentially expressed genes.

### Candidate effector genes have a distinct TE environment

According to the ‘two‐speed genome’ model, candidate effector genes tend to cluster in gene‐sparse and repeat‐rich compartments of the genome (see the Introduction). We analyzed the intergenic distances between neighboring genes in *B.g. tritici*. Indeed, candidate effector genes have an increased intergenic distance (5′‐end 20.5 kb, 3′‐end 20.4 kb) compared with noneffector genes (5′‐end 13.8 kb, 3′‐end 13.9 kb) (Fig. S16). However, the difference is not as big as reported for other species, where the difference can be one order of magnitude (Dong *et al*., 2015). Thus, we concluded that *B.g. tritici* does not contain the typical TE‐rich compartment of a ‘two‐speed’ genome.

**Figure 5:**
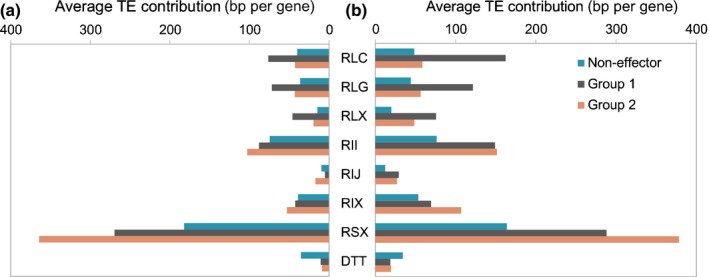
Transposable element (TE) content in flanking regions of candidate effector genes. TE composition of sequences 2 kb (a) upstream and (b) downstream of genes is shown. For each 2‐kb segment, the total cumulative contribution of different TE superfamilies was calculated for each group of genes. Values for all TE superfamilies were added up separately for group 1 and group 2 candidate effectors and noneffector genes. RLC,* Copia*; RLG,* Gypsy*; RLX, long terminal repeat retrotransposon of unknown superfamily; RII, long interspersed nuclear elements (LINEs) of the *I* superfamily; RIJ, LINEs of the *Jokey* superfamily; RIX, LINEs of unknown superfamily; RSX, short interspersed nuclear elements; DTT,* Mariner*.

Because most TEs contain promoter elements, their insertion into promoters of genes can potentially affect the expression of genes. Thus, we studied classification and transcriptional orientation of the TEs closest to genes. Here, we only considered TEs that are < 2 kb away from the start and stop codons of the coding sequence of genes (Fig. S5). Again, we surveyed group 1 and 2 effector genes and noneffector genes separately. Only 50% of the noneffector genes have TEs in the 2 kb upstream of genes (Table S20). By contrast, over 80% of group 1 and 2 effector genes do. Upstream regions of group 1 effector genes are enriched in long terminal repeat retrotransposons of the *Copia* and *Gypsy* superfamilies, whereas upstream regions of group 2 effector genes are enriched in long interspersed nuclear elements and short interspersed nuclear elements (compared with noneffector genes; Fig. 5; Table S20). Interestingly, the closest TEs upstream of genes are found in reverse orientation more often than would be expected if TEs inserted in random orientation.

Duplications and deletions of genes can be caused by unequal crossing over (UECO) between repeated sequences in their flanking regions. We found that approximately half of all genes that show CNV in the 36 *B.g. tritici* isolates also have TEs of the same family and the same orientation up‐ and downstream of the gene (Table S21). Candidate effector genes have significantly more such UECO templates than noneffector genes do. Unequal crossing over between such repeat templates resulting in duplications (or deletions) of genes that lie between the templates may be selectively advantageous for effector genes. Interestingly, only 7.6% of noneffector genes appear to be prone to CNV, although about one‐third have potential UECO templates in their flanking region. It is possible that CNV in noneffector genes is often deleterious and, therefore, selected against.

### Candidate effector genes are enriched in highly recombinogenic regions

**Figure 6:**
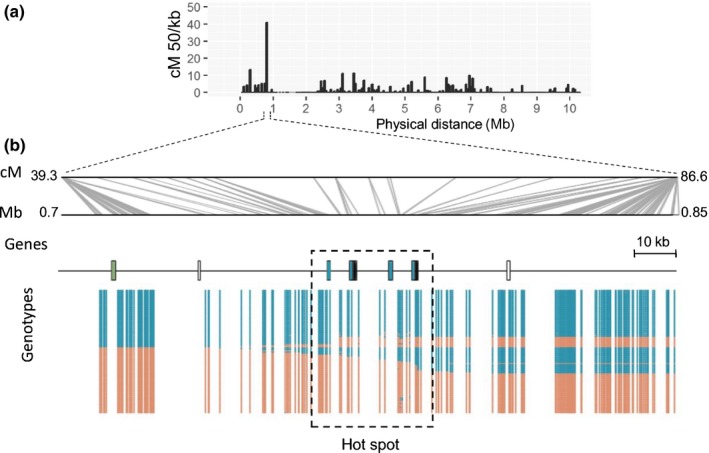
Molecular analysis of a recombination hot spot in *Blumeria graminis* f. sp. *tritici*. (a) Recombination frequency along Bgt_chr‐10 is plotted in 50‐kb windows. (b) Detailed analysis of a recombination hot spot on Bgt_chr‐10 and the flanking 50‐kb intervals. The physical organization of the hot spot is shown. Candidate effectors genes are indicated by colored boxes: green, *E014_AvrPm2* family members; blue, *E008_AvrPm3a2/f2* family members. Black indicates *WE190* family members, and empty boxes represent noneffector genes. Underneath, the genotypes of the 118 progeny are plotted, illustrating the hot spot around the *AvrPma* ^*a2/f2*^ and *WE190* gene family members. Red markers correspond to the genotype of 96224; blue corresponds to the genotype of THUN‐12.

Unequal crossover in regions with high recombination rate can contribute to gene evolution (Gaut *et al*., 2007). To test in *B.g. tritici* whether candidate effector genes are enriched in recombination‐active regions, we extracted information about the sequence content flanking 2124 recombination breakpoints in the 96224 × THUN‐12 population. These recombination breakpoints were defined as the middle nucleotide position between two consecutive recombining SNP markers that are < 4000 bp apart. In the 1 kb flanking regions of the recombination breakpoint we found 262 genes, of which 26% (67) are candidate effector genes and 74% (195) are noneffectors genes (Table S22). Thus, candidate effector genes were significantly enriched around recombination points (25% vs 10% in the genome, exact binomial test *P* < 0.05). Both group 1 and group 2 candidate effector genes were enriched. The enrichment persisted if the interval was enlarged, but it became weaker: in the interval flanking 50 kb of the recombination breakpoints, candidate effector genes represent 11.4% of the total 5698 genes. Fig. 6 shows a region of 150 kb containing the recombination hot spots on chromosome 10 flanked by two low‐recombination regions. Within the recombination hot spot there are four candidate effector genes of the *E008_AvrPm3a2/f2* family and two genes of the WE family WE090 (additional examples in Fig. S17). In many organisms, high recombination rate was associated with open chromatin structure and, consequently, increased expression. However, we did not observe an increased expression of genes that are located near recombination breakpoints compared with the whole‐genome average (Table S22). High recombination rate can also favor gene conversion events (Gaut *et al*., 2007). We therefore searched for potential gene conversion events (i.e. apparent double crossing‐over events that occurred in very small physical intervals) in the 96224 × THUN‐12 genetic map. We only considered potential conversions that are < 1 kb in size and overlap with genes. We identified seven potential gene conversion events, of which two are located in candidate effector clusters. Therefore, a small percentage (< 3%) of the recombination breakpoints found within 1 kb of genes could actually be the result of gene conversion events.

## Discussion

The genomes of many fungal and oomycete plant pathogens are characterized by a high repeat content that presumably plays a role in the adaptation to their host (Dong *et al*., 2015; Möller & Stukenbrock, 2017). Thus, a high‐quality assembly that includes repetitive sequences is necessary to improve our understanding of plant pathogen evolution. The 140.6 Mb sized chromosome‐scale assembly of *B.g. tritici* isolate 96224 presented in this study is 70% longer than the previously published fragmented genome assembly, due to longer scaffolds and improved assembly of the 85% repetitive sequences. In addition, we increased the number of annotated candidate effector genes by 40% and we could resolve many duplicated genes. Only by including a high‐resolution genetic map for scaffolding were we able to determine chromosome number, assemble the 11 *B.g. tritici* chromosomes end to end, and determine location, size and sequence organization of centromeres and the highly repetitive rDNA loci.

### Recombination is associated with effector content and chromosomal location

In sexually reproducing organisms, recombination is an important driver for genetic diversity (Gaut *et al*., 2007; Möller & Stukenbrock, 2017), as it can create new gene combinations, eliminate deleterious mutations, and accelerate the rate of selection by breaking linkage disequilibrium. In addition, it can favor structural rearrangements through UECO and thus contribute to CNV (Gaut *et al*., 2007). In most organisms, recombination is unevenly distributed along chromosomes, and thus the effects of recombination events vary (Jensen‐Seaman *et al*., 2004; Gaut *et al*., 2007; Choi & Henderson, 2015; Mezard *et al*., 2015). In yeast, recombination occurs most often in G/C‐rich regions in the central regions of chromosome arms (Gerton *et al*., 2000). By contrast, in *B. graminis*, the regions adjacent to the centromeres are enriched in recombination hot spots, but we found no association of recombination with G/C content. In other fungal plant pathogens, such as *Zymoseptoria tritici* and *Fusarium graminearum*, recombination tends to occur near telomeres (Croll *et al*., 2015; Laurent *et al*., 2018). We suggest that this unequal distribution of recombination leads to variable rates of new gene combinations along the chromosomes.

Interestingly, we found that candidate effector genes are enriched around recombination breakpoints. Studies in *Z. tritici* and *F. graminearum* also found such an association (Croll *et al*., 2015; Laurent *et al*., 2018). We suggest that close proximity of effector genes to recombination breakpoints can drive the creation of new combinations of candidate effector genes within gene clusters and contribute to cluster expansion. However, our data also indicate that gene conversion (which is a nonreciprocal exchange of DNA) could in part counteract the effector gene diversification.

It is unclear to what degree the enrichment around recombination contributes to gene evolution in natural populations, since little is known about the conservation of recombination frequencies between different crosses. Indeed, in *Z. tritici*, recombination hot spots have been shown to be only partially conserved between mapping populations (Croll *et al*., 2015). Here, we found that recombination hot and cold spots are conserved between three genetic crosses sharing the same parental isolate 96224. However, the *Z. tritici* mapping populations did not share parental strains. It is therefore possible that the presence of one conserved parent in crosses is sufficient to define hot and cold spots of recombination.

### Duplications, CNV and varying expression levels drive diversification of candidate effector genes

Candidate effector genes evolve rapidly and through multiple mechanisms. First, candidate effector genes are more often duplicated than noneffector genes are in our reference mildew isolate 96224. These duplications predominantly occur as tandem or inverted tandem repeats, and thus contribute to cluster formation. The high level of sequence similarity between duplicated genes indicates that they arose recently in evolution. Moreover, we found duplicated candidate effector genes that were transferred to different chromosomes where they can potentially found new clusters. Duplications are crucial for gene evolution, allowing one copy to evolve freely while the other copy retains the original function, or allowing both genes to diversify independently. Identification of these identical or highly similar gene copies critically depended on a high‐quality genome assembly, since such duplicated genes are often collapsed in fragmented genome assemblies (S. Bourras *et al*., unpublished).

Second, our analysis of 36 *B.g. tritici* isolates showed that candidate effectors are more prone to duplication and deletion than noneffector genes are. Extensive CNV in candidate effector gene families between different ff. ssp. of *Blumeria graminis* was described previously (Menardo *et al*., 2017). Here, we show that > 14% of the candidate effectors also display CNV within the same f. sp. A similar level of CNV was found in 135 small secreted proteins in *Z. tritici* (Hartmann & Croll, 2017). In addition to driving rapid evolutionary divergence, gene duplications can contribute to dosage effects. For example, we found that isolate 94202, which is highly virulent on wheat lines carrying different *Pm3* alleles (Bourras *et al*., 2015), encodes at least three copies of the suppressor of avirulence *SvrPm3*. Consequently, we found very high expression for this gene in 94202. Conversely, gene loss can help adaptation by removing nonbeneficial gene variants from the genome (Sanchez‐Vallet *et al*., 2018). This is exemplified by the absence of *BgtE‐8545* (*AvrPm2*) in many isolates (Praz *et al*., 2017), which makes these isolates virulent on wheat lines carrying the resistance gene *Pm2*. Our findings share similarities with those from smut fungi, where candidate effectors were also found to evolve through duplications and deletions of genes within clusters (Dutheil *et al*., 2016). Smut fungi are basidiomycetes. Thus, mechanisms by which effector genes evolve may be very similar even in distantly related taxa.

Third, we observed a wide range of gene expression levels among genes from the same candidate effector gene clusters. Bourras *et al*. (2018) proposed that low‐expressed effector genes are maintained as ‘latent’ genes in the genome and serve as a ‘reservoir’ for genetic variation. Such latent genes may spontaneously be activated and selected for if they are beneficial to the pathogen. Indeed, we found that candidate effector genes are more often differentially expressed between three isolates than noneffector genes are, suggesting that regulation of effector repertoire is a common mechanism.

Interestingly, unlike in typical ‘two‐speed’ genomes (Dong *et al*., 2015), we found no evidence for higher nucleotide substitution rates in candidate effector genes, which is consistent with the loss of the RIP pathway (Wicker *et al*., 2013; Spanu, 2017). In the absence of a mechanism that increases mutation rates, we propose that high levels of CNV and transcriptional diversity could be mechanisms that contribute to maintaining mildew adaptability to its host. Together, these mechanisms maintain a highly dynamic candidate effector gene repertoire in individual wheat powdery mildew isolates.

### Group 1 effector genes show highest variability in gene content and transcription

Although candidate effector genes have a higher plasticity than noneffector genes (see the Results section), this plasticity is not equal between gene families. Group 1 candidate effector gene families show higher levels of expression and CNV than group 2 candidate effector genes. Additionally, group 1 genes contain more members that are differentially expressed, and they have higher ratios of nonsynonymous to synonymous substitution rates. Interestingly, Pedersen *et al*. (2012) found that group 1 homologues from *B.g. hordei* are predominantly expressed in the haustorium, whereas longer candidate effectors are universally expressed both in the haustorium and the hyphal tissue. Haustorium formation is a critical step in the establishment of infection and probably requires well‐adapted effectors to properly manipulate the host cell. In addition, many of the race‐specific plant immune receptors are supposedly located in the cytoplasm. We propose that these factors put strong selection pressure on candidate effectors that are predominantly active at this important stage during infection. Interestingly, the four avirulence effectors *AvrPm2*,* AvrPm3a2/f2*,* AvrPm3b2/c2* and *AvrPm3d3* and the suppressor of avirulence (*SvrPm3*) are all group 1 genes (Bourras *et al*., 2015, unpublished; Praz *et al*., 2017). To avoid recognition, avirulence genes accumulate mutations that disrupt the recognition by the *R* gene (McNally *et al*., 2018). However, avoidance of recognition may also interfere with effector protein function. We suggest that the high number of gene duplications leads to functional redundancy, allowing the gene copies to diversify independently, and therefore might compensate for this effect.

### *Blumeria g. tritici* genome provides an environment for rapid gene evolution

The recent study on the barley powdery mildew genome by Frantzeskakis *et al*. (2018) proposed a ‘one‐speed’ genome, compared with the ‘two‐speed’ genome hypothesis for plant pathogens (Dong *et al*., 2015). As in *B.g. hordei*, candidate effectors in *B.g. tritici* are not localized in specific genomic compartments that are typical for a two‐speed genome. Instead, individual candidate effector gene families cluster along chromosomes and are intermingled with noneffector genes. Nevertheless, genomic ‘environments’ of group 1 and 2 effector genes and noneffector genes differ from each other in their distinct TE compositions.

We found that many *B.g. tritici* genes have TEs of the same family in the same orientation in their flanking regions. Such direct repeats can serve as templates for UECO and thus to gene duplication or deletion. However, templates for UECO were found in similar numbers around group 1 and group 2 candidate effector genes as well as noneffector genes, but CNV was most often found in group 1 candidate effectors. This suggests that the high TE content of *B.g. tritici* provides the templates for CNV, but gene duplications are only maintained if they provide a selective advantage. Similarly, group 1 genes seem to tolerate TE insertions into promoters better than group 2 and noeffector genes do. UECO between TEs has also been suggested as a driver of CNV in smut fungi (Dutheil *et al*., 2016).

In summary, our findings indicate that candidate effector genes indeed evolve at a higher ‘speed’ than noneffector genes do. However, the increased pace of evolution is not a result of genomic compartmentalization. Instead, we propose that the highly repetitive nature of the powdery mildew genome provides mechanisms for a fast evolution of all genes, but that the actual pace of evolution of individual effector genes is determined by the degree of positive selection on them.

## Author contributions

BK, TW, CRP and MCM planned and designed the research, interpreted results, and wrote the manuscript. MCM and CRP contributed equally. CRP, MCM, TW, FM, AGS, LK, SS, SO, MP, AW and SB performed experiments. CRP, MCM, TW and AGS analyzed data. MCM and CRP contributed equally to this work.

## Supporting information

Please note: Wiley Blackwell are not responsible for the content or functionality of any Supporting Information supplied by the authors. Any queries (other than missing material) should be directed to the New Phytologist Central Office.

**Fig. S1** Linear regression between chromosome size and gene numbers per chromosome.
**Fig. S2** Contributions to the *B.g. tritici* genome of the 20 most abundant TE families.
**Fig. S3** Distribution of GC content in genomic windows with different recombination frequencies.
**Fig. S4** Identification and characterization of tandem repeats that were collapsed in the PacBio assembly.
**Fig. S5** Sequence organization of the centromere of chromosomes 8 and 11.
**Fig. S6** BUSCO assessment of the *B.g. tritici* genome version v3.16.
**Fig. S7** General characteristics of recombination in the cross 96224 × THUN‐12.
**Fig. S8** PCR amplification of genomic regions acting as recombination hotspots in both parental isolates.
**Fig. S9** Physical clustering of effector gene families along *B.g. tritici* chromosomes 1–11.
**Fig. S10** Phylogenetic and expression analyses of candidate effector gene families E007 and E011.
**Fig. S11** Gene expression in the 16 candidate effector families with > 10 genes.
**Fig. S12** Expression levels of the 16 largest candidate effector gene families in 96224.
**Fig. S13** Distribution of normalized genomic coverage for selected genes in 36 *B.g. tritici* isolates.
**Fig. S14** Heat map of normalized genomic coverage of candidate effector family E014.
**Fig. S15** Amino acid compositions of 7164 predicted Non‐effector proteins, 412 group 1 and 243 group 2 candidate effector proteins.
**Fig. S16** Two‐speed‐genome hypothesis tested in *B.g. tritici*.
**Fig. S17** Example of recombination hotspots in the 96224 × THUN‐12 population.
**Methods S1** Construction of the genetic map.
**Methods S2**Transcriptome analysis.
**Methods S3** Phylogenetic analyses.
**Methods S4** Statistical analyses.
**Notes S1** Verification of the integrity of the genome assembly in recombination hotspots by PCR.
**Notes S2** Analysis of sequence diversity of genes in 36 *B.g. tritici* isolates.
**Notes S3** A chromosome‐scale assembly of the *B.g. tritici* genome.
**Notes S4** Genome size estimation.
**Notes S5** Transposable element annotation.
**Notes S6** Gene annotation and candidate effector classification.
**Table S1** Sizes and features of the assembled chromosomes.
**Table S2** Size estimates of five collapsed repeats.
**Table S3** *Blumeria graminis tritici1* isolates used for analyses of centromeric repeats, mapping coverage and copy number variations.
**Table S4** Other *formae speciales* isolates used for analyses of centromeric repeats and mapping coverage.
**Table S5** Presence of centromeric tandem repeats *CentA* and *CentB* in various *Blumeria graminis formae speciales*.
**Table S6** Numbers of homologs of candidate effector genes and non‐effector genes in the two Leotiomycetes *Botrytis cinerea* and *Phialocephala subalpina*.
**Table S7** Summary of the genetic information for the 11 chromosomes of *B.g. tritici* from the cross 96224 × THUN‐12.
**Table S8** Description of the recombination hotspots that were validated by PCR as described in Notes S5.
**Table S9** Primers used to verify the recombination hotspots by PCR.
**Table S10** Recombination frequency in the 96224 × THUN‐12 depending of the genomic origin of THUN‐12.
**Table S11** Conservation of recombination hotspots in three mapping populations.
**Table S12** Conservation of recombination coldspots in three mapping populations.
**Table S13** Description of the 16 candidate effector gene families with at least 10 members in *B.g. tritici*.
**Table S14** Summary of duplicated genes in the reference assembly Bgt_genome.v3.16.
**Table S15** Copy number variation of genes in 36 isolates of *B.g. tritici*.
**Table S16** Estimates of nucleotide polymorphism rates in synonymous sites of genes derived from sequences of 36 *B.g. tritici* isolates.
**Table S17** Nucleotide polymorphism rates in *B.g. tritici* genes from 36 isolates.
**Table S18** Nucleotide polymorphism rates in *B.g. tritici* genes from 36 isolates.
**Table S19** Enrichment of candidate effector among differentially expressed genes.
**Table S20** Enriched TE superfamilies in up‐ and downstream regions of group 1 and 2 candidate effector genes as well as non‐effector genes.
**Table S21** Number of copy number variants (CNV) and templates for unequal crossing over (UECO).
**Table S22** Expression levels of candidate effector and non‐effector genes located near recombination breakpoints.
