# Supplementary material for: A chromosome‐scale genome assembly reveals a highly dynamic effector repertoire of wheat powdery mildew
Source: New Phytol. 2018 Nov 14;221(4):2176–89. doi: 10.1111/nph.15529 (PMC6587952; doi:10.1111/nph.15529)
Supplement: Supplementary file 1 — Fig. S1 Linear regression between chromosome size and gene numbers per chromosome. Fig. S2 Contributions to the B.g. tritici genome of the 20 most abundant TE families. Fig. S3 Distribution of GC content in genomic windows with different recombination frequencies. Fig. S4 Identification and characterization of tandem repeats that were collapsed in the PacBio assembly. Fig. S5 Sequence organization of the centromere of chromosomes 8 and 11. Fig. S6 BUSCO assessment of the B.g. tritici genome version v3.16. Fig. S7 General characteristics of recombination in the cross 96224 × THUN‐12. Fig. S8 PCR amplification of genomic regions acting as recombination hotspots in both parental isolates. Fig. S9 Physical clustering of effector gene families along B.g. tritici chromosomes 1–11. Fig. S10 Phylogenetic and expression analyses of candidate effector gene families E007 and E011. Fig. S11 Gene expression in the 16 candidate effector families with > 10 genes. Fig. S12 Expression levels of the 16 largest candidate effector gene families in 96224. Fig. S13 Distribution of normalized genomic coverage for selected genes in 36 B.g. tritici isolates. Fig. S14 Heat map of normalized genomic coverage of candidate effector family E014. Fig. S15 Amino acid compositions of 7164 predicted Non‐effector proteins, 412 group 1 and 243 group 2 candidate effector proteins. Fig. S16 Two‐speed‐genome hypothesis tested in B.g. tritici. Fig. S17 Example of recombination hotspots in the 96224 × THUN‐12 population. Methods S1 Construction of the genetic map. Methods S2Transcriptome analysis. Methods S3 Phylogenetic analyses. Methods S4 Statistical analyses. Notes S1 Verification of the integrity of the genome assembly in recombination hotspots by PCR. Notes S2 Analysis of sequence diversity of genes in 36 B.g. tritici isolates. Notes S3 A chromosome‐scale assembly of the B.g. tritici genome. Notes S4 Genome size estimation. Notes S5 Transposable element annotation. Notes S6 Gene annotation and candid [file NPH-221-2176-s001.pdf]

***New Phytologist* Supporting Information**

Article title: **A chromosome-scale genome assembly reveals a highly dynamic effector repertoire of wheat powdery mildew**

Authors: Marion C. Müller* , Coraline R. Praz*, Alexandros G. Sotiropoulos, Fabrizio Menardo, Lukas Kunz, Seraina Schudel, Simone Oberhänsli, Manuel Poretti, Andreas Wehrli, Salim Bourras, Beat Keller, Thomas Wicker

*These authors contributed equally to this work

Article acceptance date: 02 October 2018

The following Supporting Information is available for this article:

Fig. S1 Linear regression between chromosome size and gene numbers per chromosome.

Fig. S2 Contributions to the *B.g. tritici* genome of the 20 most abundant TE families.

Fig. S3 Distribution of GC content in genomic windows with different recombination frequencies.

Fig. S4 Identification and characterization of tandem repeats that were collapsed in the PacBio assembly.

Fig. S5 Sequence organization of the centromere of chromosomes 8 and 11.

Fig. S6 BUSCO assessment of the *B.g. tritici* genome version v3.16.

Fig. S7 General characteristics of recombination in the cross 96224 X THUN-12.

Fig. S8 PCR amplification of genomic regions acting as recombination hotspots in both parental isolates.

Fig. S9 Physical clustering of effector gene families along *B.g. tritici* chromosomes 1-6.

Fig. S9 (continued). Physical clustering of effector gene families along *B.g. tritici* chromosomes 7-11.

Fig. S10 Phylogenetic and expression analyses of candidate effector gene families E007 and E011.

Fig. S11 Gene expression in the 16 candidate effector families with more than 10 genes.

Fig. S12 Expression levels of the 16 largest candidate effector gene families in 96224.

Fig. S13 Distribution of normalized genomic coverage for selected genes in 36 *B.g. tritici* isolates.

Fig. S14 Heat map of normalized genomic coverage of candidate effector family E014.

Fig. S15 Amino acid compositions of 7,164 predicted Non-effector proteins, 412 group 1 and 243 group 2 candidate effector proteins.

Fig. S16 Two-speed-genome hypothesis tested in *B.g. tritici.*

Fig. S17 Example of recombination hotspots in the 96224 X THUN-12 population.

Table S1 Sizes and features of the assembled chromosomes.

Table S2 Size estimates of five collapsed repeats.

Table S3 *B.g. tritici1* isolates used for analyses of centromeric repeats, mapping coverage and copy number variations.

Table S4 Other *formae speciales* isolates used for analyses of centromeric repeats and mapping coverage.

Table S5 Presence of centromeric tandem repeats *CentA* and *CentB* in various *Blumeria graminis formae speciales.*

Table S6 Numbers of homologs of candidate effector genes and non-effector genes in the two Leotiomycetes *Botrytis cinerea* and *Phialocephala subalpina*

Table S7 Summary of the genetic information for the 11 chromosomes of *B.g. tritici* from the cross 96224 X THUN-12.

Table S8 Description of the recombination hotspots that were validated by PCR as described in Note S1.

Table S9 Primers used to verify the recombination hotspots by PCR.

Table S10 Recombination frequency in the 96224 X THUN-12 depending of the genomic origin of THUN-12.

Table S11 Conservation of recombination hotspots in three mapping populations.

Table S12 Conservation of recombination coldspots in three mapping populations.

Table S13 Description of the 16 candidate effector gene families with at least 10 members in *B.g. tritici*.

Table S14 Summary of duplicated genes in the reference assembly Bgt_genome.v3.16

Table S15 Copy number variation of genes in 36 isolates of *B.g. tritici*.

Table S16 Estimates of nucleotide polymorphism rates in synonymous sites of genes derived from sequences of 36 *B.g. tritici* isolates.

Table S17 Nucleotide polymorphism rates in *B.g. tritici* genes from 36 isolates.

Table S18 Nucleotide polymorphism rates in *B.g. tritici* genes from 36 isolates.

Table S19 Enrichment of candidate effector among differentially expressed genes.

Table S20 Enriched TE superfamilies in up- and downstream regions of group 1 and 2 candidate effector genes as well as non-effector genes.

Table S21 Number of copy number variants (CNV) and templates for unequal crossing over (UECO).

Table S22 Expression levels of candidate effector and non-effector genes located near recombination breakpoints.

Method S1 Construction of the genetic map.

Method S2 Transcriptome analysis.

Method S3 Phylogenetic analyses.

Method S4 Statistical analyses.

Note S1 Verification of the integrity of the genome assembly in recombination hotspots by PCR.

Note S2 Analysis of sequence diversity of genes in 36 *B.g. tritici* isolates.

Note S3 A chromosome-scale assembly of the *B.g. tritici* genome.

Note S4 Genome size estimation.

Note S5 Transposable element annotation.

Note S6 Gene annotation and candidate effector classification.

Fig. S1 Linear regression between chromosome size and gene numbers per chromosome. The x-axis is chromosome size in Mb, while the y-axis indicates the number of genes per chromosome. Individual chromosomes are indicated with squares. **a.** Overall, the total number of genes and chromosome size correlate strongly with a Pearson product-moment correlation coefficient (PMCC) of r=0.978 (When r is close to 1, it indicates a strong positive relationship). **b,** Numbers of effectors correlate less well, but still strongly, with chromosome. **c.** Numbers of group 1 and 2 candidate effectors (in total 657 genes) do not correlate with chromosome size. This is possibly due to the strong physical clustering of gene families observed in these two groups. **d.** In contrast, numbers of candidate effectors that were not included in group 1 or 2 correlate strongly with chromosome size.

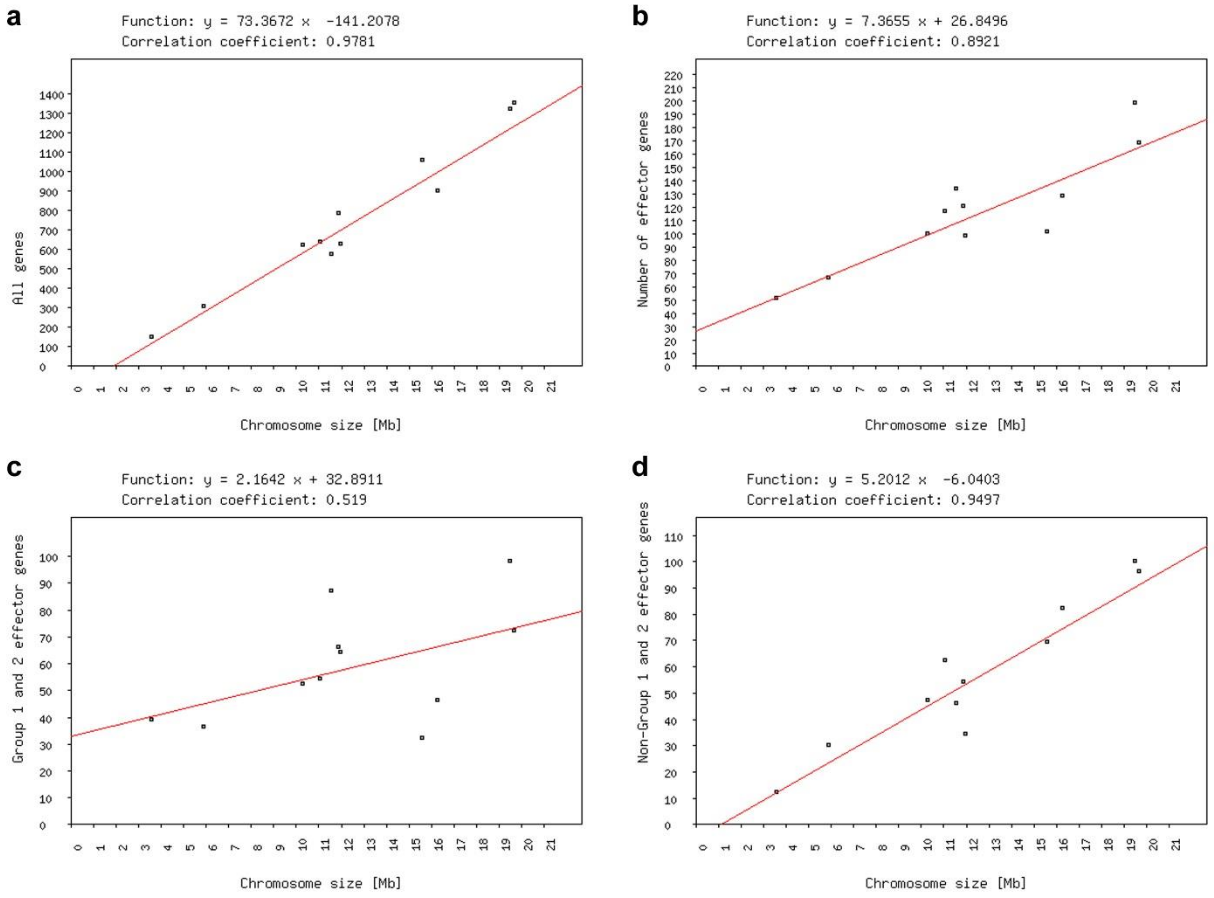

Fig. S2 Contributions to the *B.g. tritici* genome of the 20 most abundant TE families TE family names are given at the left. The second column indicates the size of the consensus sequence of the TE family. The copy number estimate is calculated by dividing the total number of bp contributed by the TE family by the size of the consensus sequence. The x-axis of the graph shows the contribution in % of the total genome size. Different superfamilies are shown in different colors.

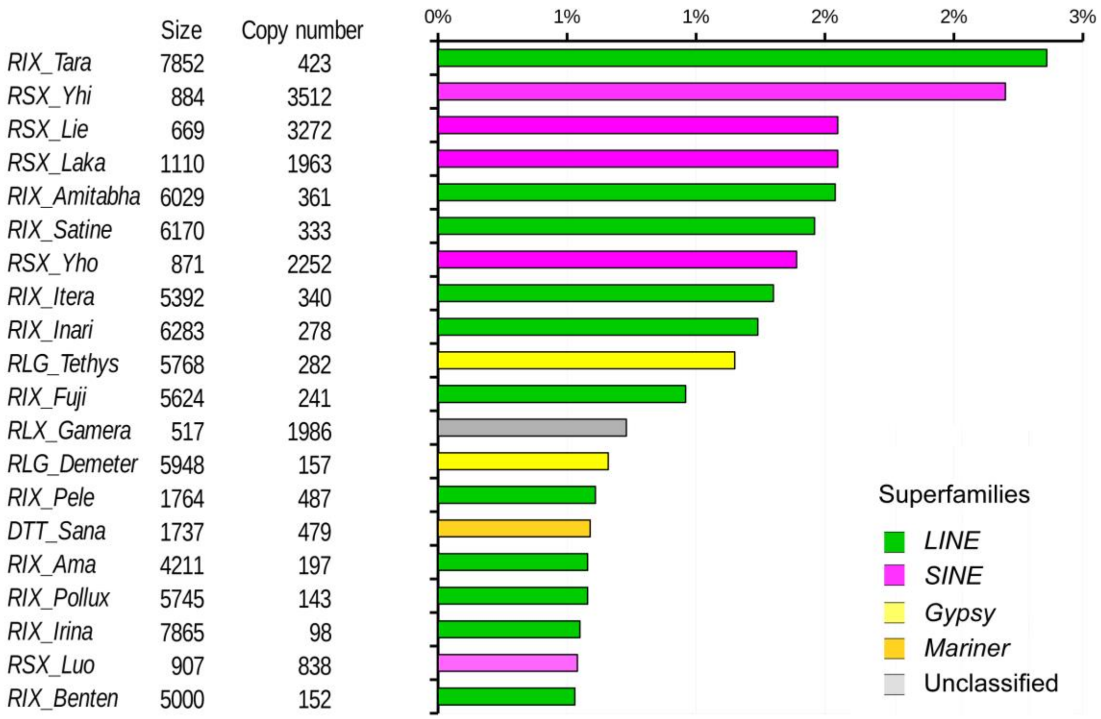

Fig. S3 Distribution of GC content in genomic windows with different recombination frequencies. GC content and recombination was estimated in 50kb windows. Windows in which no recombination occurred (cM=0) were compared to windows in with recombination (cM>0 or cM>5cM). Centromeric windows consist of windows that completely overlap with the defined centromeres (Fig. 2).

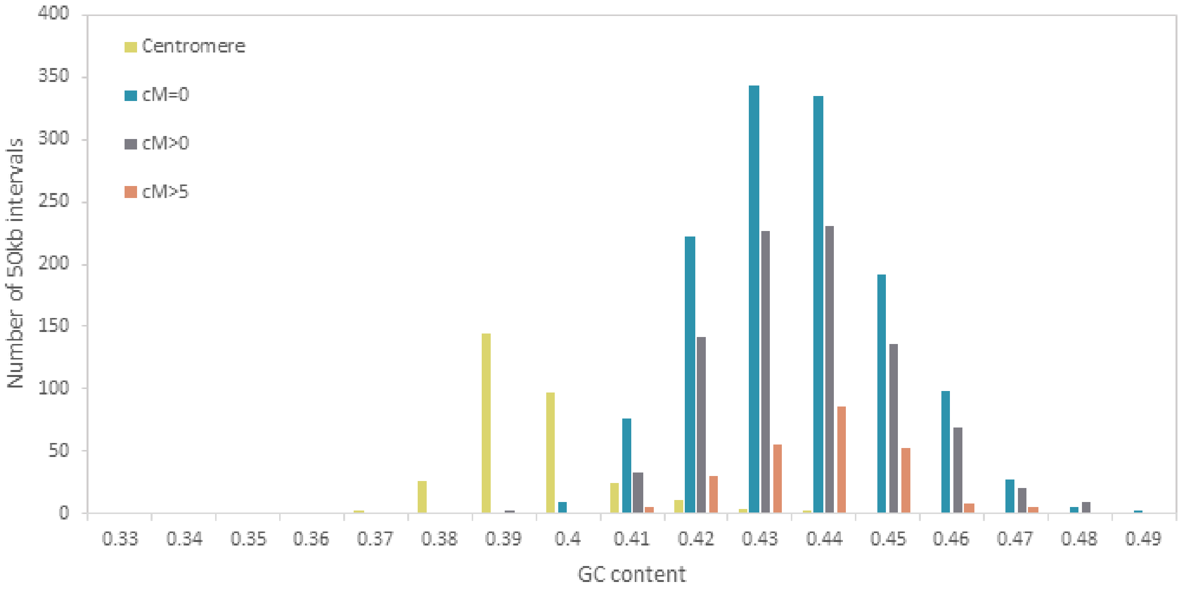

Fig. S4 Identification and characterization of tandem repeats that were collapsed in the PacBio assembly. a, To identify collapsed repeats, Illumina sequences were mapped to the *B.g. tritici* pseudomolecules. The inset at the top right depicts the distribution of sequence coverage in 500 bp windows, showing that Illumina sequences cover the genome approximately 23-fold. Sequence coverage is plotted along chromosomes in vertical red lines. The scale is in Mb. Most of the chromosomes have the expected even sequence coverage of approximately 23-fold. Regions containing collapsed repeats are visible as spikes. Most sequences representing collapsed repeats are deposited in Chr-Un. DRC represent a newly identified tandem repeat (see Note S4). **b,** Dotplot of a region containing CentA repeats. **c,** Example of a PacBio contig which shows extreme variation in G/C content. The x-axis represents the bp position while the y-axis shows the G/C content in sliding windows of 200 bp.

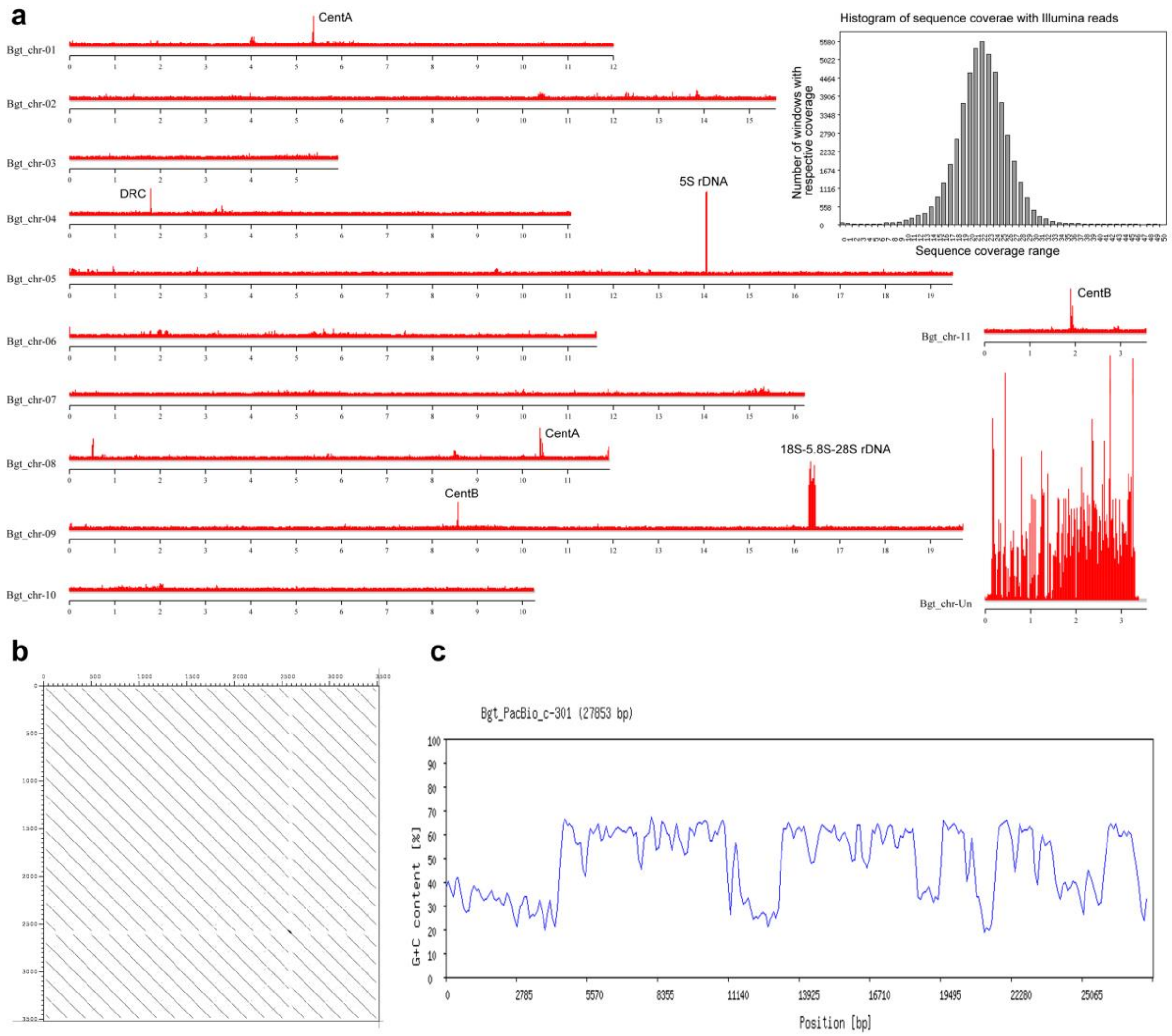

Fig. S5 Sequence organization of the centromere of chromosomes 8 and 11. **a**, Sequence coverage with Illumina reads in the 1 Mb region containing the centromere of chromosome 8. **b**, Sequence coverage around the region containing centromeric repeats type A in chromosome 8. In **a** and **b**, the dark grey boxes indicate regions with higher coverage corresponding to the centromeric repeats type A (*CentA*); the x-axis corresponds to positions over the chromosome in bp and the y-axis to the coverage in number of Illumina reads. **c**, Annotation of the region corresponding to **b**. **d**, Annotation of the centromere of chromosome 8. **e**, Annotation of the centromere of chromosome 11. The legend box corresponds to **c**, **d** and **e**.

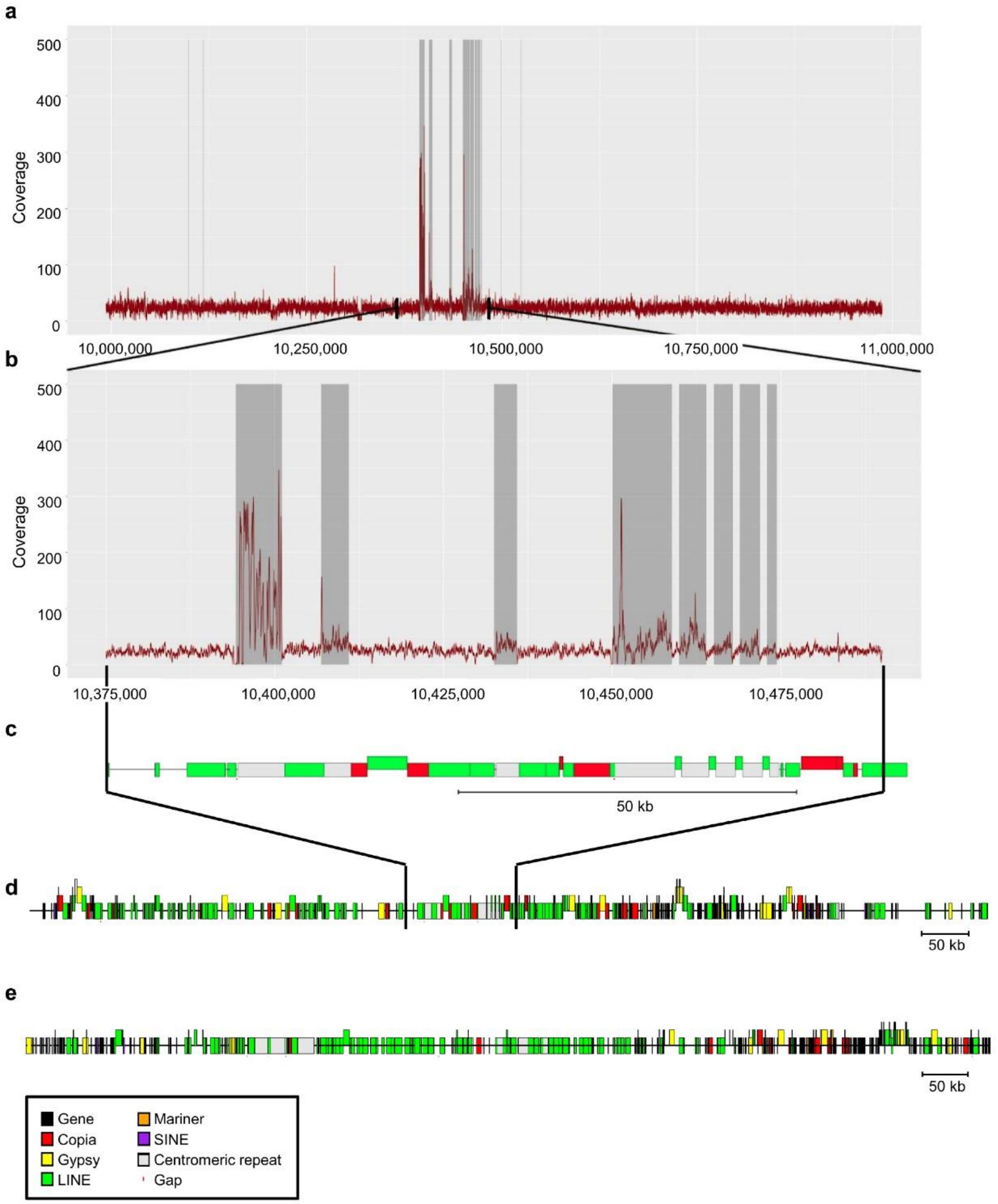

Fig. S6 BUSCO assessment of the *B.g. tritici* genome version v3.16. BUSCO was done with gene sets for the taxa Ascomycetes and fungi. The dataset for fungi is smaller (290 genes) and contains more highly conserved genes of which almost all (286) are present in the *B.g. tritici* genome version v3.16. The higher number of absent genes in Ascomycetes is due to the larger number of genes used in that dataset (1275), and the fact that the reference species of the database is *Aspergillus nidulans*, which is a rather distant relative of *B.g. tritici.*

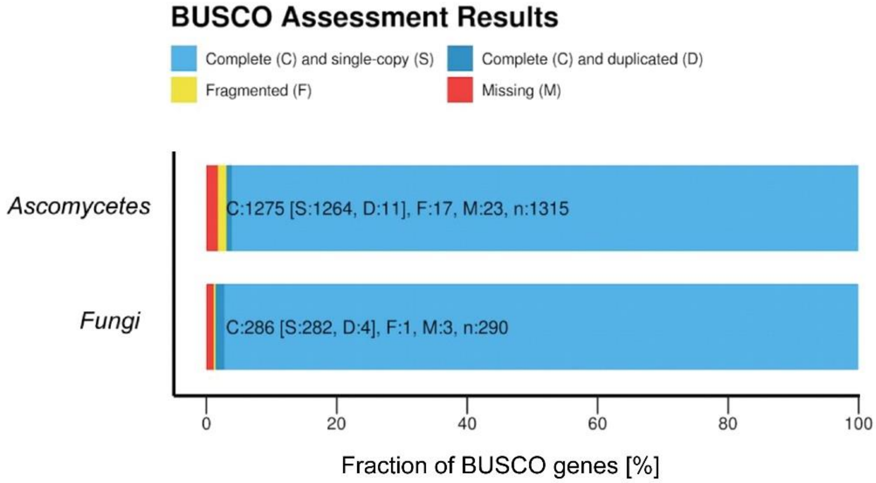

Fig. S7 General characteristics of recombination in the cross 96224 X THUN-12. a, Distribution of the physical distance of 1,520 marker pairs that are genetically >1cM apart from each other **b,** Correlation between chromosome size and recombination rate. Every dot represents one chromosome. **c,** Correlation between chromosome size and size of the centromeric regions. Each dot represents one chromosome. **d,** Distribution of recombination rate estimated in 50kb windows.

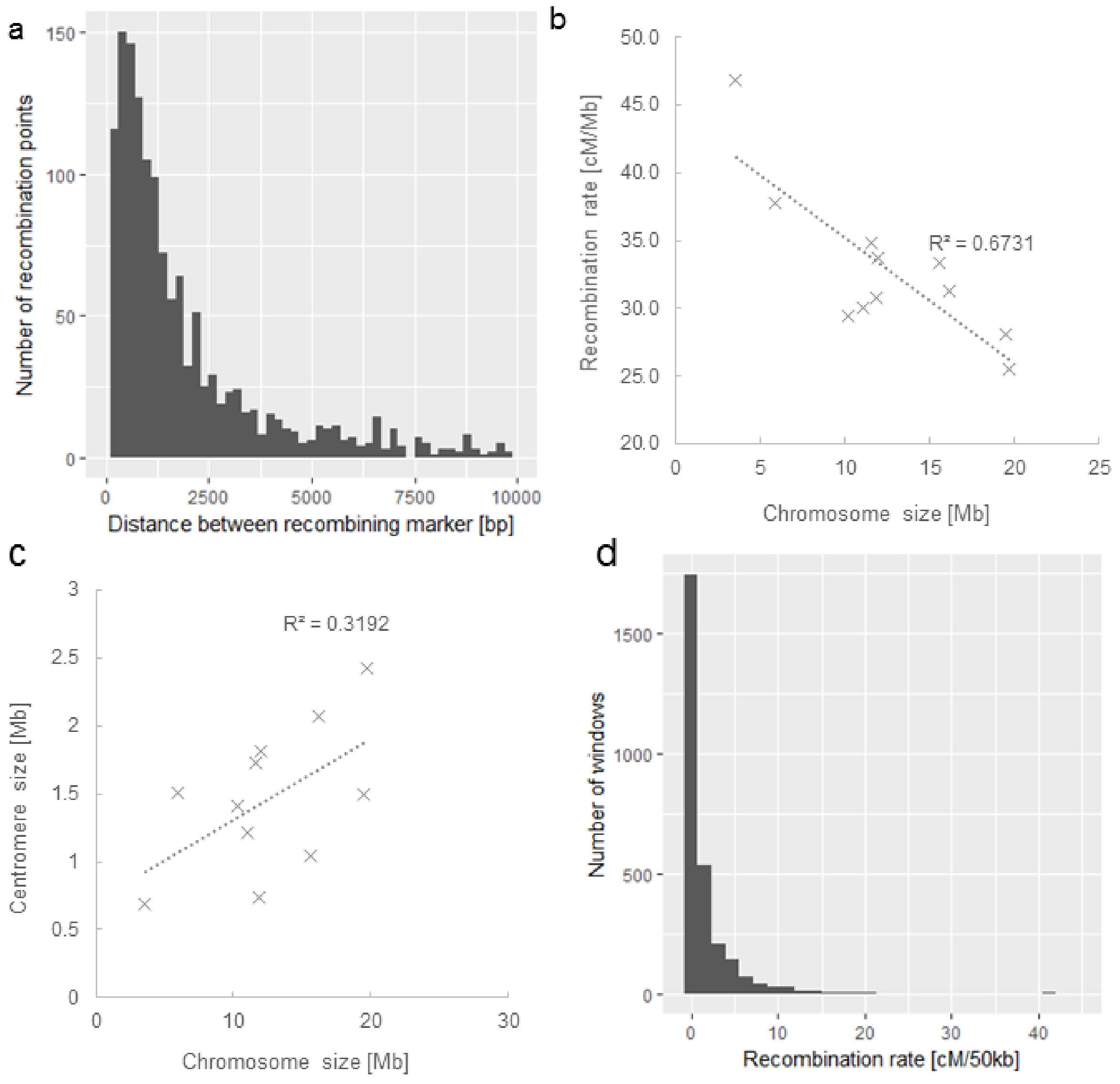

Fig. S8 PCR amplification of genomic regions acting as recombination hotspots in both parental isolates. Recombination hotspots are described in Supplementary Table 7. Each hotspot region was amplified in 96224 (left band) and THUN-12 (right band). **a**. Hotspots RH1-RH8 **b**. Hotspots RH9-RH18

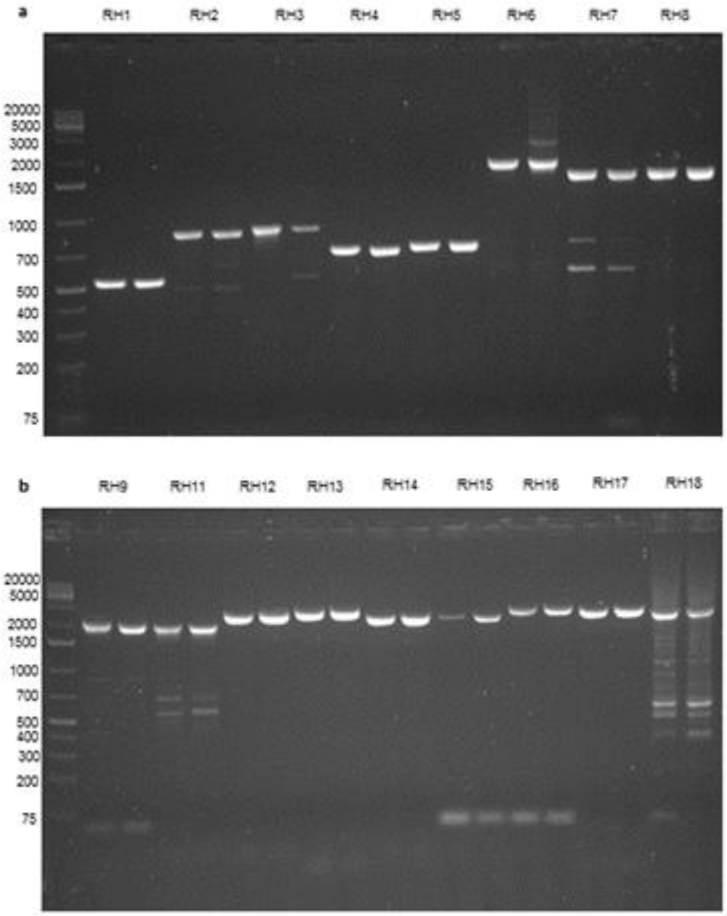

Fig. S9 Physical clustering of effector gene families along *B.g. tritici* chromosomes 1-6. The physical positions of *B.g. tritici* genes are plotted along their chromosomal position (x-axis, in kb). Each dot represents one gene. In the lowest row in grey all genes are plotted. The second row, in black, represents all candidate effector genes and the next 21 rows represent the 21 candidate effector gene families containing more than 10 members.

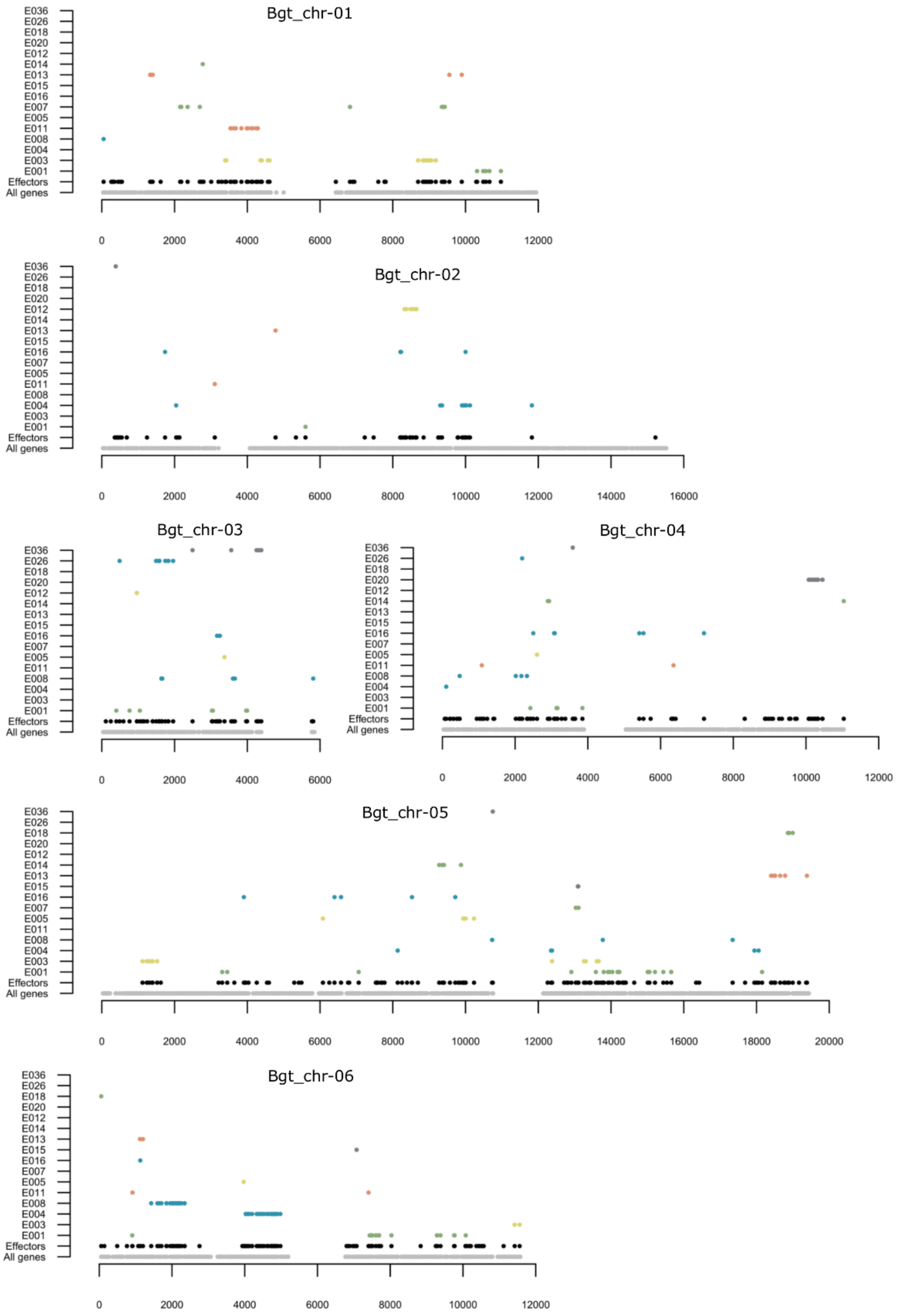

Fig. S9 (continued) Physical clustering of effector gene families along *B.g. tritici* chromosomes 7-11. The physical positions of *B.g. tritici* genes are plotted along their chromosomal position (x-axis, in kb). Each dot represents one gene. In the lowest row in grey all the genes are plotted. The second row, in black, represents all candidate effector genes and the next 21 rows represent the 21 candidate effector gene families containing more than 10 members.

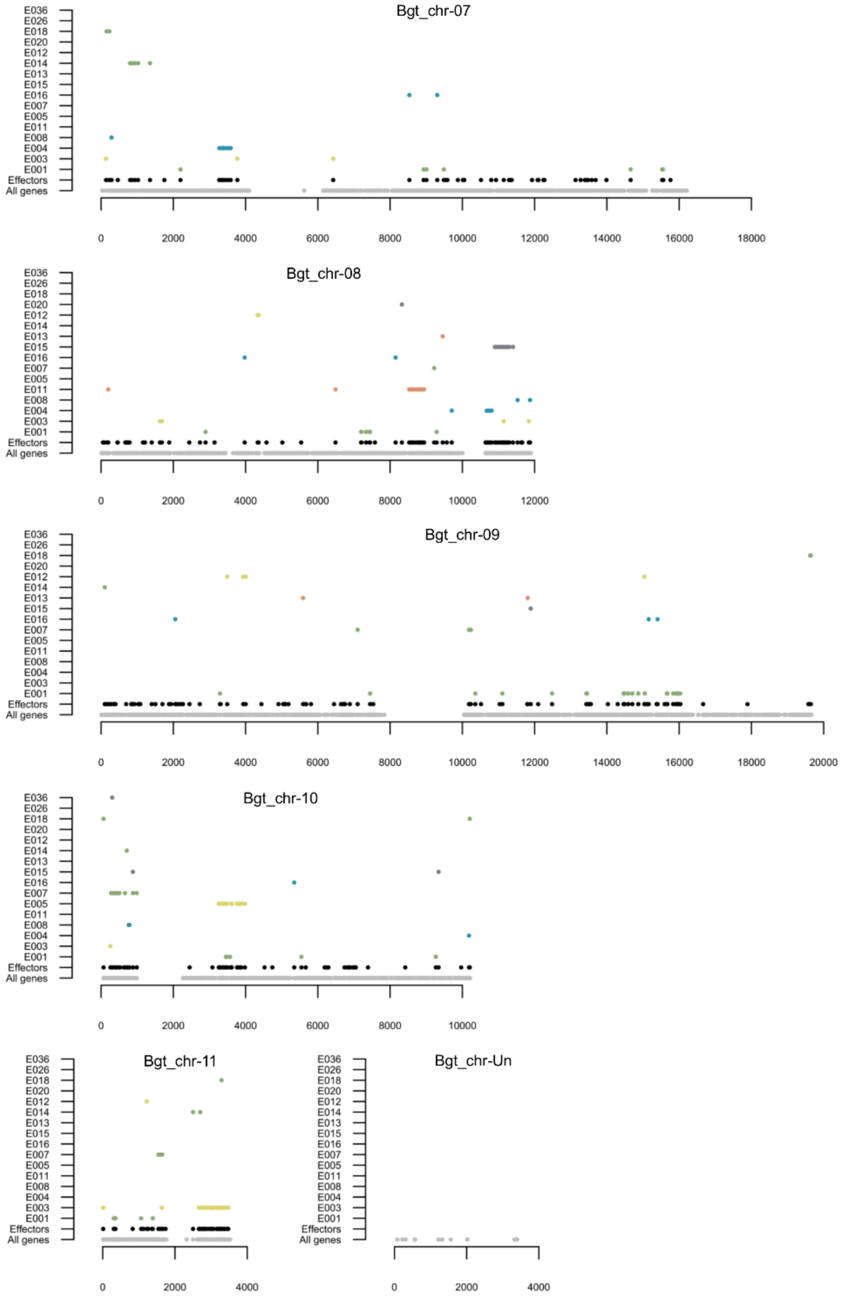

Fig. S10 Phylogenetic and expression analyses of candidate effector gene families E007 and E011. a. Phylogenetic tree of effector gene family E007. **b.** Gene expression of the members of family E007. **c.** Phylogenetic tree of effector gene family E011. **d.** Gene expression of the members of family E011. **e.** Physical position of the six clusters of effector family E007 and two clusters of family E001. The different clusters are indicated on their chromosomal positions with boxes of different colors corresponding to the colors in **a**, **b**, **c** and **d** respectively. For **a** and **c**, the grey branches correspond to *B.g. hordei* genes and the *B.g. tritici* genes are indicated either with colored branches corresponding to different clusters or with black branches for genes that are not part of a cluster. For **b** and **d**, the expression values are indicated in rpkm based on three biological replicates and the error bars represent the standard error of the mean. The colors refer to the clusters in **a** and **c** respectively. Gene expression varies drastically between family members as well as between genes of the same cluster.

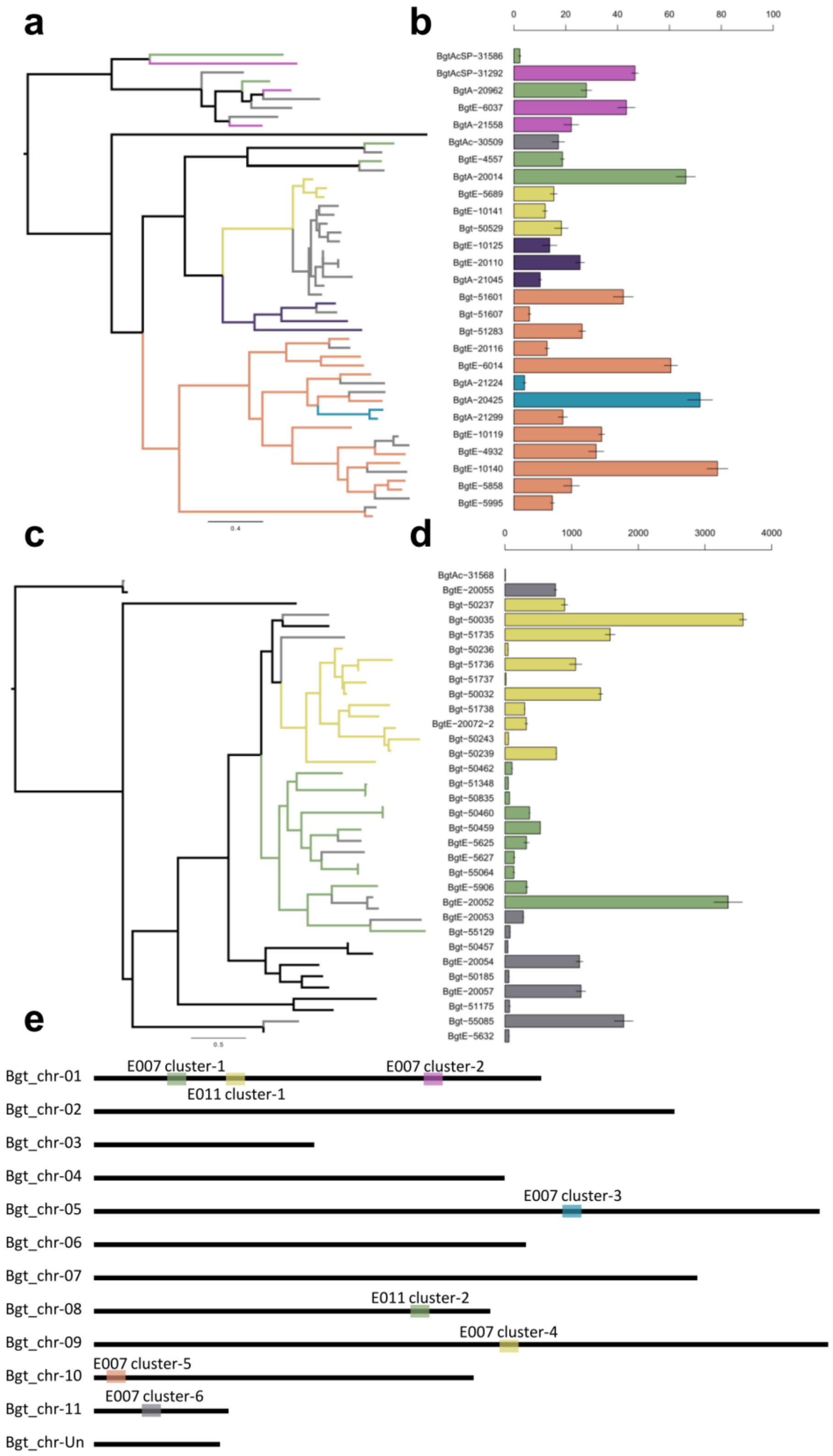
a
b
0
20
40
60
80
100
BgtAcSP-31586
BgtAcSP-31292
BgtA-20962
BgtE-6037
BgtA-21558
BgtAc-30509
BgtE-4557
BgtA-20014
BgtE-5689
BgtE-10141
Bgt-50529
BgtE-10125
BgtE-20110
BgtA-21045
Bgt-51601
Bgt-51607
Bgt-51283
BgtE-20116
BgtE-6014
BgtA-21224
BgtA-20425
BgtA-21299
BgtE-10119
BgtE-4932
BgtE-10140
BgtE-5858
BgtE-5995
0.4
c
d
0
1000
2000
3000
4000
BgtAc-31568
BgtE-20055
Bgt-50237
Bgt-50035
Bgt-51735
Bgt-50236
Bgt-51736
Bgt-51737
Bgt-50032
Bgt-51738
BgtE-20072-2
Bgt-50243
Bgt-50239
Bgt-50462
Bgt-51348
Bgt-50835
Bgt-50460
Bgt-50459
BgtE-5625
BgtE-5627
Bgt-55064
BgtE-5906
BgtE-20052
BgtE-20053
Bgt-55129
Bgt-50457
BgtE-20054
Bgt-50185
BgtE-20057
Bgt-51175
Bgt-55085
BgtE-5632
0.5
e
Bgt_chr-01
E007 cluster-1
E007 cluster-2
E011 cluster-1
Bgt_chr-02
Bgt_chr-03
Bgt_chr-04
Bgt_chr-05
E007 cluster-3
Bgt_chr-06
Bgt_chr-07
Bgt_chr-08
E011 cluster-2
Bgt_chr-09
E007 cluster-4
Bgt_chr-10
E007 cluster-5
Bgt_chr-11
E007 cluster-6
Bgt_chr-Un

Fig. S11 Gene expression in the 16 candidate effector families with more than 10 genes. Gene expression levels of all members of the candidate effector gene families with more than 10 genes are plotted in reads per kilo basepairs per million reads (rpkm). Mean expression of three RNA-Seq biological replicates is plotted and the error bars represent standard deviation of the mean. The genes are ordered by increasing expression levels. Families in the green frame are from group-1, the ones in the blue frame from group-2 and the family in the yellow frame is family E016, the only family with more than 10 members that could not be attributed to one of the two groups.

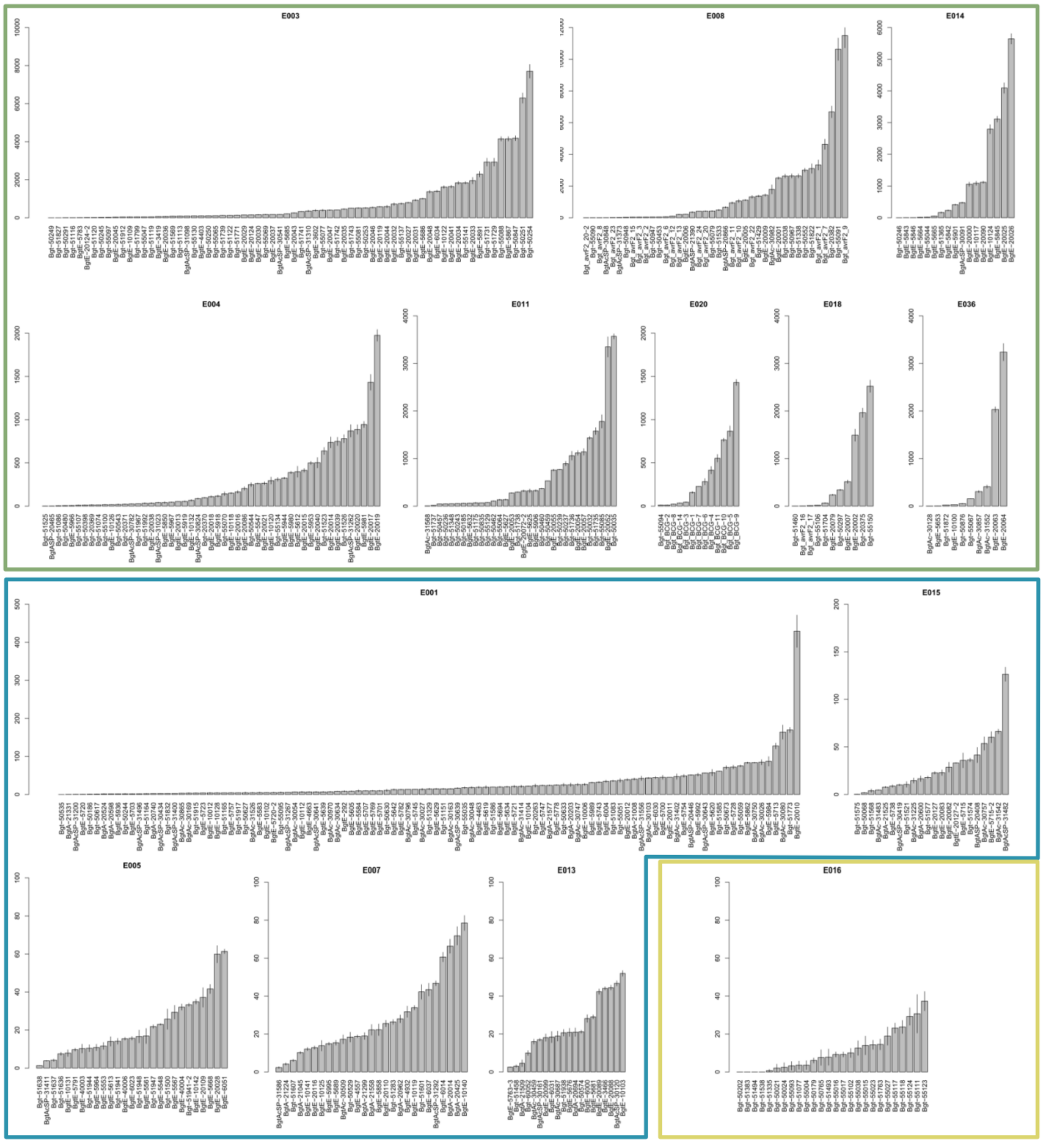

Fig. S12 Expression levels of the 16 largest candidate effector gene families in 96224. The expression level was deduced from three RNA-Seq replicates and depicted in log(rpkm). Gene families of group 1 candidate effectors are depicted in green, families of group 2 in blue and family E016 in yellow.

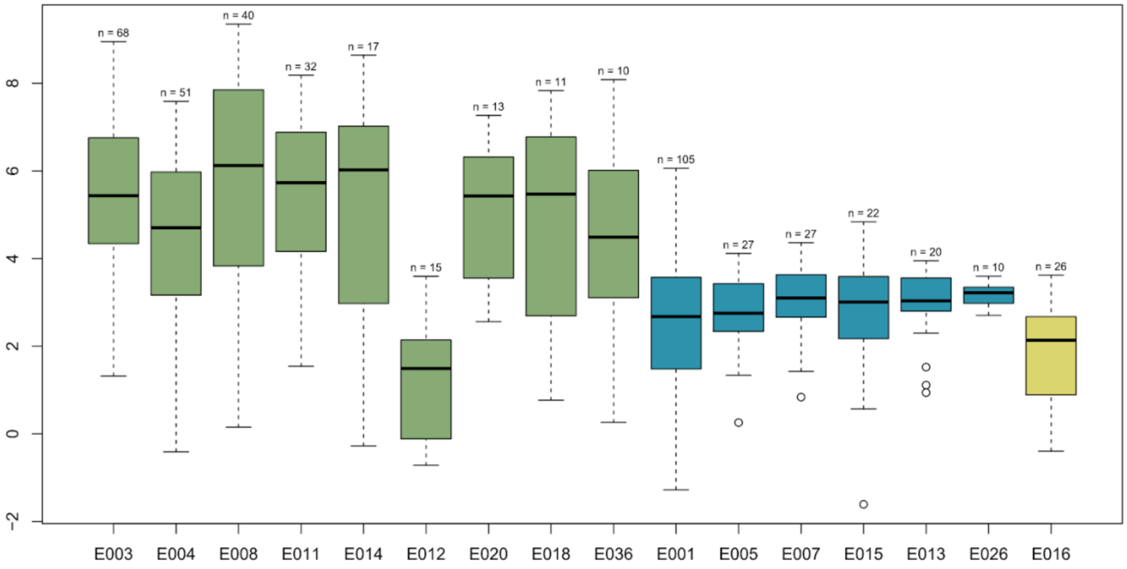

Fig. S13 Distribution of normalized genomic coverage for selected genes in 36 *B.g. tritici* isolates. Genomic coverage for each gene was normalized to the coverage of all genes in the genome. The first six genes are non-effector genes that served as control- Bgt-712 = GAPDH, Bgt-715 = β-Tubulin, Bgt-940 = α-Tubulin, Bgt-1043 = monoglyceride lipase, Bgt-1486 = Actin, Bgt-1778 = H3 Histone. The next six genes are candidate effector genes. BgtE-5845 = AvrPm2 from family E014, Bgt_BCG-1 = SvrPm3 from family E020, Bgt_avrF2_7 = AvrPm3$^{a2/f2}$ from family E008, BgtAc-30757 = family E015 member, BgtE-20015 = E004 family member, Bgt-51636 = family E005 member.

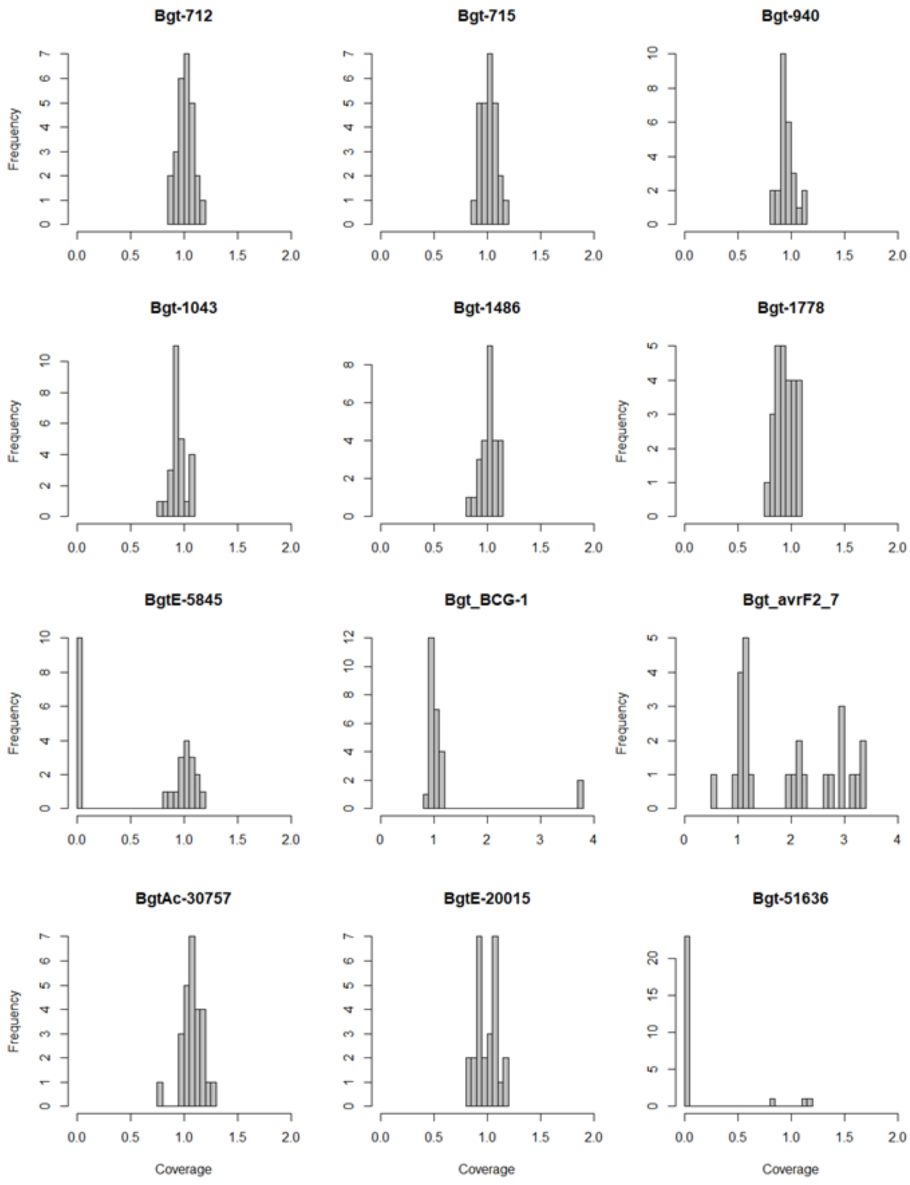

Fig. S14 Heat map of normalized genomic coverage of candidate effector family E014. Genomic coverage for each gene was normalized to the coverage of all genes. The genes are ordered according to the phylogenetic tree in Fig. 3. The isolates originate from Switzerland (CH), The United Kingdom (UK), China (CHN) and Israel (ISR).

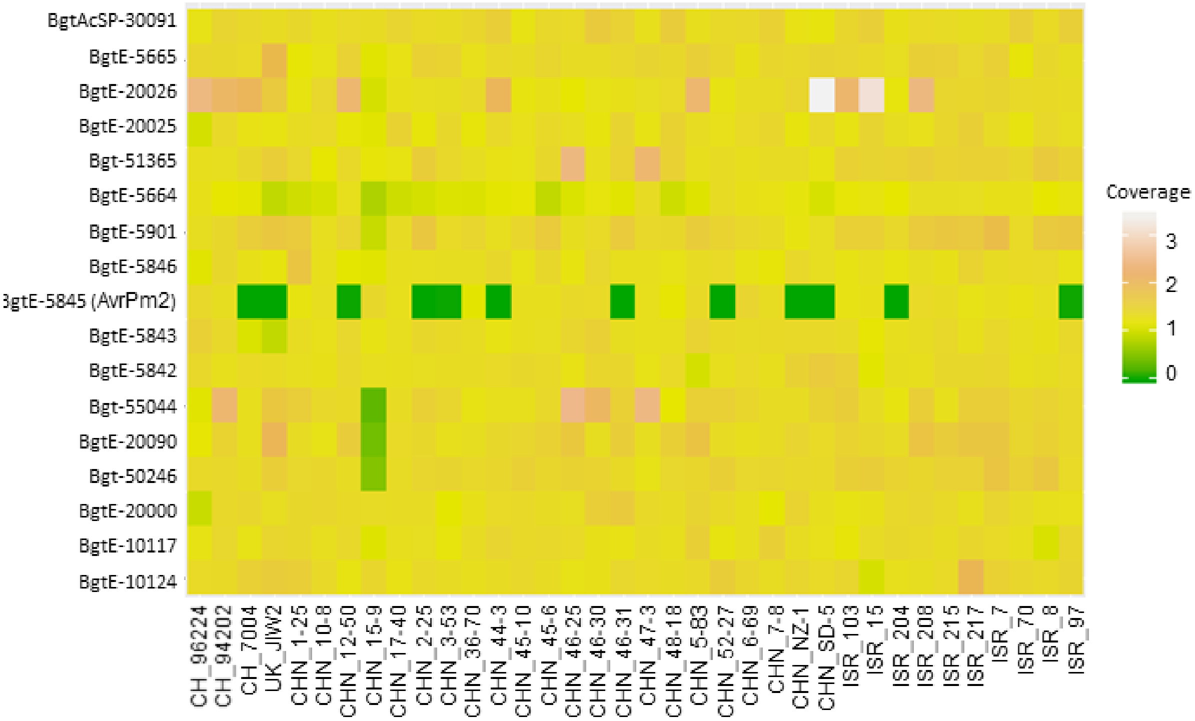

Fig. S15 Amino acid compositions of 7,164 predicted Non-effector proteins, 412 group 1 and 243 group 2 candidate effector proteins. Multiple Amino-acids that are encoded by multiple codons (Alanine, Leucine, Arginine and Serine) are under-represented in group 1 and 2 candidate effector proteins, while amino acids with only two codons (Cysteine, Phenylalanine and Tyrosine) are over-represented. This amino acid usage bias contributes to the higher number of synonymous sites in non-effector genes.

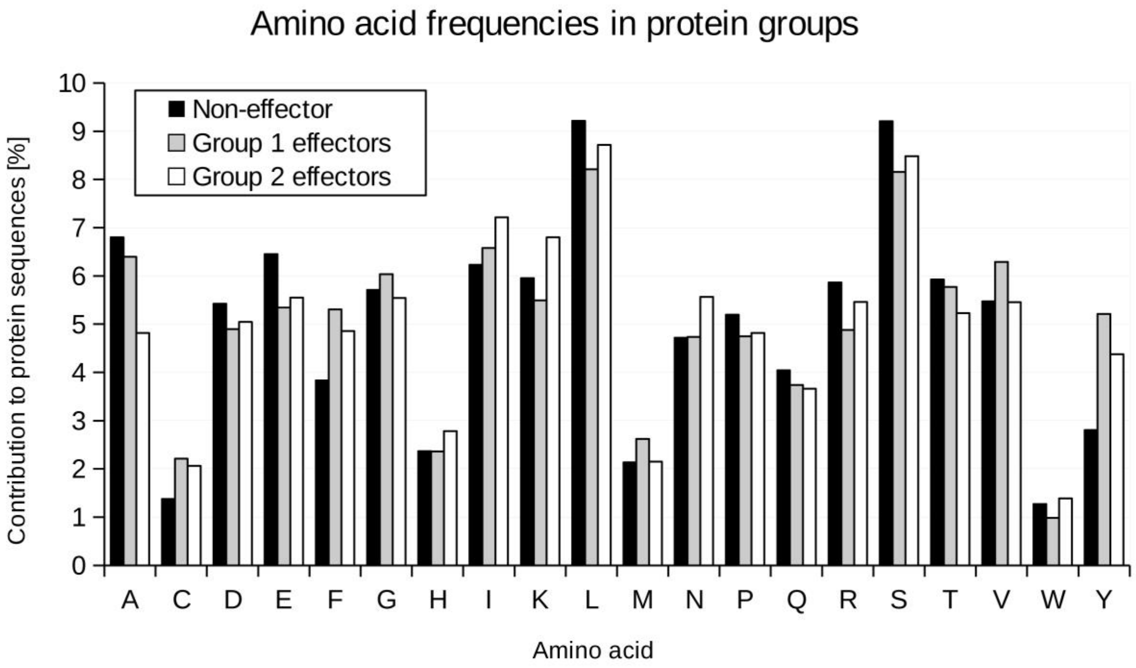

Fig. S16 Two-speed-genome hypothesis tested in *B.g. tritici.* Density plots displaying the intergenic distance for each gene from the 3'UTR and 5'UTR of the coding sequence. **a**, shows all 8,470 genes. **b**, 844 candidate effector genes. Red dots indicate known avirulence genes (*Avr*) and the suppressor of avirulence (*Svr)* in *B.g. tritici.*

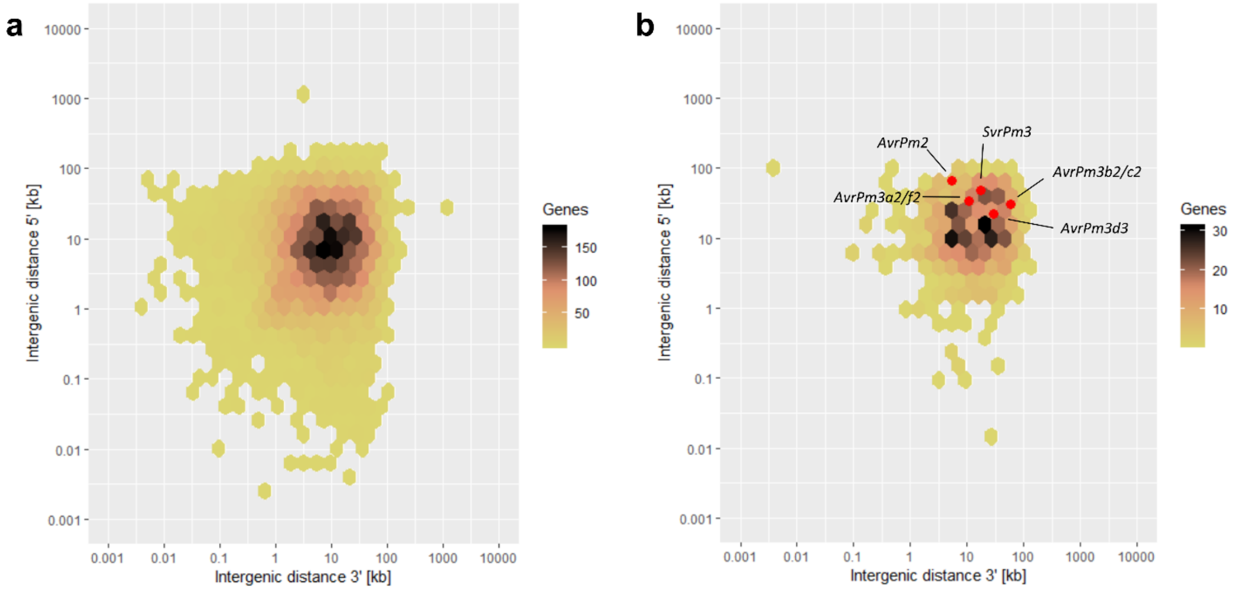

Fig. S17 Example of recombination hotspots in the 96224 X THUN-12 population. For each hotspot a physical distance of 150 kb is shown. Upper panels show collinearity between genetic (upper line) and physical (lower line) position of the SNP marker in the interval. Middle panels show the genes localized in the interval. Candidate effector genes of families with more than 10 members are indicated in color, effector candidate genes belonging to families with less than 10 members in black and non-effector genes by empty boxes. The third panel shows the genotypes of the 118 progeny for each SNP marker. Blue color indicates 96224 genotype and red indicates THUN-12 genotype. Grey color indicates missing data for the SNP marker. **a,** Bgt_chr-01 3350000-3500000, red: candidate effector family E003, black: candidate effector family E060 **b,** Bgt_chr-01 40500000-42000000, black: candidate effector family E046 **c,** Bgt_chr-04 2350000-2500000 green: candidate effector family E001 , blue: candidate effector family E016, black: candidate effector family E148 **d,** Bgt_chr-06 4900000-5050000 blue: candidate effector family E004

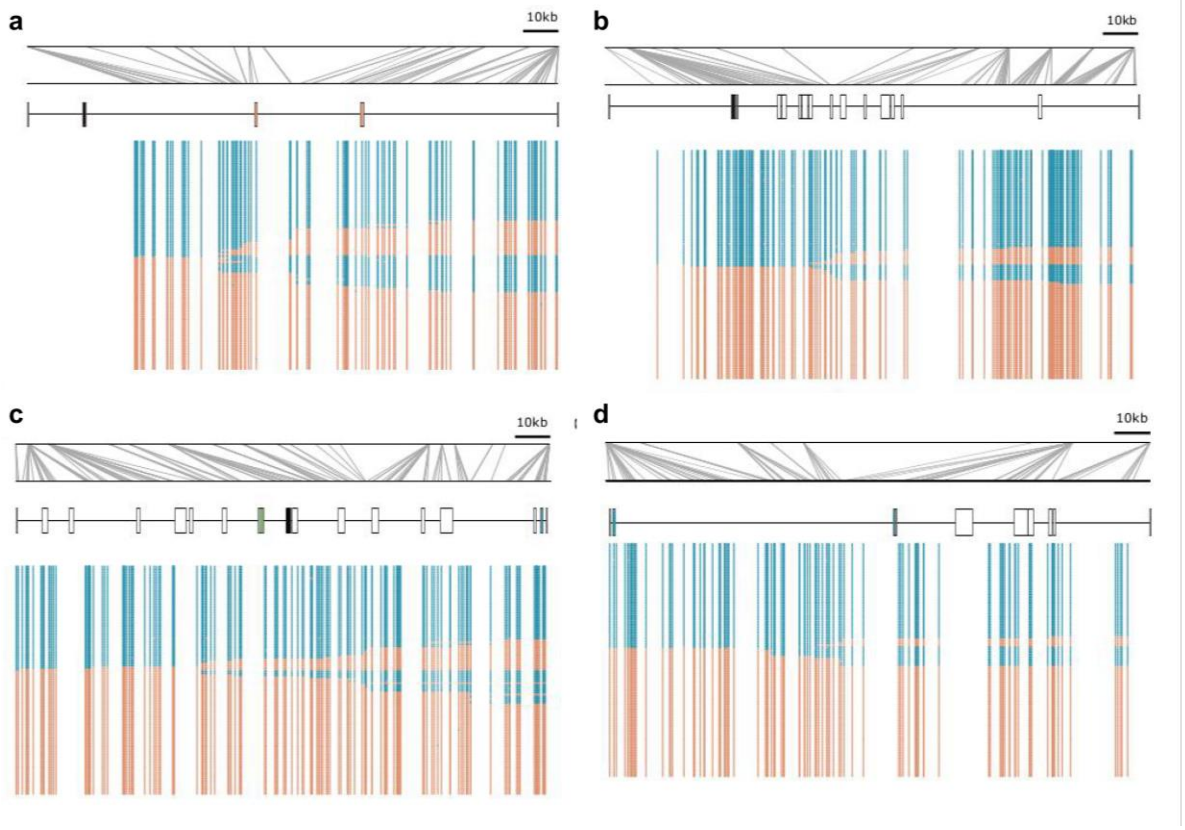

Table S1 **Sizes and features of the assembled chromosomes.**

Chromosome	Size[1]	Sequence gaps[2]	Genes[3]	Candidate effectors[4]
Bgt_chr-01	12.01	29	638	72
Bgt_chr-02	15.59	48	1070	49
Bgt_chr-03	5.93	13	318	46
Bgt_chr-04	11.07	29	644	75
Bgt_chr-05	19.48	42	1,330	131
Bgt_chr-06	11.62	41	584	98
Bgt_chr-07	16.22	39	914	65
Bgt_chr-08	11.9	26	795	86
Bgt_chr-09	19.72	50	1362	107
Bgt_chr-10	10.25	30	633	69
Bgt_chr-11	3.57	10	161	46
Bgt_chr-Un	3.4	3395	21	-
Total	140.8	357	8,470	844

[1] Size of the chromosomes is indicated in Mb.
[2] Number of sequence gaps per chromosome.
[3] Number of genes annotated per chromosome.
[4] Number of annotated candidate effector genes per chromosome.

Table S2 **Size estimates of five collapsed repeats.**

Repeat	In assembly [kb][1]	Extrapolated size [kb][2]
CentA	65	440
CentB	14	76
DRC	133	1,156
rDNA 45S	474	5,823
rDNA 5S	343	3,742
TE hit	143	435
No hit	1,063	14,178
Total	1,172	25,850

[1] Cumulative length of the repeat type in the PacBio assembly.

[2] Extrapolated length, calculated from Illumina sequence coverage.

Table S3 ***B.g. tritici*** **isolates used for analyses of centromeric repeats, mapping coverage and copy number variations.**

Isolate	Forma specialis	Published in[1]	Accession Number	BLAST[2]	Mapping[3]	CNV[4]
7	*B.g. tritici*	Menardo *et* al., 2016	SRP062198	*	*	*
8	*B.g. tritici*	Menardo et al. 2016	SRP062198	*	*	*
15	*B.g. tritici*	Menardo et al. 2016	SRP062198	*	*	*
70	*B.g. tritici*	Menardo et al. 2016	SRP062198	*	*	*
97	*B.g. tritici*	Menardo et al. 2016	SRP062198	*	*	*
103	*B.g. tritici*	Menardo et al. 2016	SRP062198	*	*	*
204	*B.g. tritici*	Menardo et al. 2016	SRP062198	*	*	*
208	*B.g. tritici*	Menardo et al. 2016	SRP062198	*	*	*
215	*B.g. tritici*	Menardo et al. 2016	SRP062198	*	*	*
217	*B.g. tritici*	Menardo et al. 2016	SRP062198	*	*	*
7004	*B.g. tritici*	Menardo et al. 2016	SRP062198	*	*	*
94202	*B.g. tritici*	Menardo et al. 2016	SRP062198	*	*	*
JIW2	*B.g. tritici*	Wicker et al. 2013	SRP062198	*	*	*
1-25	*B.g. tritici*	Praz et al. 2017	SRP062198		*	*
2-25	*B.g. tritici*	Praz et al. 2017	SRP062198		*	*
46-30	*B.g. tritici*	Praz et al. 2017	SRP062198		*	*
46-31	*B.g. tritici*	Praz et al. 2017	SRP062198		*	*
48-18	*B.g. tritici*	Praz et al. 2017	SRP062198		*	*
3-53	*B.g. tritici*	Praz et al. 2017	SRP062198		*	*
5-83	*B.g. tritici*	Praz et al. 2017	SRP062198		*	*
6-69	*B.g. tritici*	Praz et al. 2017	SRP062198		*	*
7-8	*B.g. tritici*	Praz et al. 2017	SRP062198		*	*
10-8	*B.g. tritici*	Praz et al. 2017	SRP062198		*	*
12-50	*B.g. tritici*	Praz et al. 2017	SRP062198		*	*
NZ-1	*B.g. tritici*	Praz et al. 2017	SRP062198		*	*
17-40	*B.g. tritici*	Praz et al. 2017	SRP062198		*	*
44-3	*B.g. tritici*	Praz et al. 2017	SRP062198		*	*
45-10	*B.g. tritici*	Praz et al. 2017	SRP062198		*	*
46-25	*B.g. tritici*	Praz et al. 2017	SRP062198		*	*
SD-5	*B.g. tritici*	Praz et al. 2017	SRP062198		*	*
36-70	*B.g. tritici*	Praz et al. 2017	SRP062198		*	*
45-6	*B.g. tritici*	Praz et al. 2017	SRP062198		*	*
47-3	*B.g. tritici*	Praz et al. 2017	SRP062198		*	*
52-27	*B.g. tritici*	Praz et al. 2017	SRP062198		*	*
15-9	*B.g. tritici*	Praz et al. 2017	SRP062198		*	*

[1] Publication where the genome sequences were published

[2] Genome assemblies of the isolates marked with an * were used for the identification of centromeric repeats *CentA* and *CentB* by BLAST searches.

[3] Sequencing reads of the isolates marked with an * were mapped against the Bgt_genome_v3_16 for coverage analyses.

[4] Sequencing reads of the isolates marked with an * were used for the analysis of copy number variation (CNV) of genes

Table S4 Other *formae speciales* isolates used for analyses of centromeric repeats and mapping coverage

Isolate	Forma	Published in[1]	Accession Number	BLAST[2]	Mapping[3]
58	*B.g. tritici2*	Menardo et al. 2016	SRP062198	*	*
63	*B.g. tritici2*	Menardo et al. 2016	SRP062198	*	*
66	*B.g. tritici2*	Menardo et al. 2016	SRP062198	*	*
207	*B.g. tritici2*	Menardo et al. 2016	SRP062198	*	*
209	*B.g. tritici2*	Menardo et al. 2016	SRP062198	*	*
220	*B.g. tritici2*	Menardo et al. 2016	SRP062198	*	*
BAH-2	*B.g. triticale*	Menardo et al. 2016	SRP062198		*
BU-18	*B.g. triticale*	Menardo et al. 2016	SRP062198		*
CAP-39-A1	*B.g. triticale*	Menardo et al. 2016	SRP062198		*
COPP-2C	*B.g. triticale*	Menardo et al. 2016	SRP062198		*
HO-101	*B.g. triticale*	Menardo et al. 2016	SRP062198		*
T1-20	*B.g. triticale*	Menardo et al. 2016	SRP062198	*	*
T1-23	*B.g. triticale*	Menardo et al. 2016	SRP062198	*	*
T1-8	*B.g. triticale*	Menardo et al. 2016	SRP062198		*
T3-16	*B.g. triticale*	Menardo et al. 2016	SRP062198		*
T3-4	*B.g. triticale*	Menardo et al. 2016	SRP062198		*
T3-8	*B.g. triticale*	Menardo et al. 2016	SRP062198		*
T3-9	*B.g. triticale*	Menardo et al. 2016	SRP062198	*	*
T4-19	*B.g. triticale*	Menardo et al. 2016	SRP062198		*
T4-20	*B.g. triticale*	Menardo et al. 2016	SRP062198		*
T4-6	*B.g. triticale*	Menardo et al. 2016	SRP062198		*
T4-7	*B.g. triticale*	Menardo et al. 2016	SRP062198	*	*
T5-12	*B.g. triticale*	Menardo et al. 2016	SRP062198		*
T5-13	*B.g. triticale*	Menardo et al. 2016	SRP062198		*
T5-14	*B.g. triticale*	Menardo et al. 2016	SRP062198		*
T5-9	*B.g. triticale*	Menardo et al. 2016	SRP062198		*
T6-6	*B.g. triticale*	Menardo et al. 2016	SRP062198		*
THUN-12	*B.g. triticale*	Menardo et al. 2016	SRP062198		*
B. graminis dactylidis	*B.g. dactylidis*	Menardo et al. 2017	SRP062198	*	
S-1201	*B.g. secalis*	Menardo et al. 2016	SRP062198	*	*
S-1203	*B.g. secalis*	Menardo et al. 2016	SRP062198	*	*
S-1391	*B.g. secalis*	Menardo et al. 2016	SRP062198	*	*
S-1400	*B.g. secalis*	Menardo et al. 2016	SRP062198	*	*
S-1459	*B.g. secalis*	Menardo et al. 2016	SRP062198	*	*
DH-14	*B.g. hordei*	Spanu et al. 2010	GCA_000151065.1	*	
B. graminis avenae	*B.g. avenae*	Menardo *et* al., 2017	SRP062198	*	
B. graminis on Lolium	*B.g. lolii*	Menardo et al. 2017	SRP062198	*	
B. graminis poae	*B.g. poae*	Menardo et al. 2017	SRP062198	*	

[1] Publication where the genome sequences were published

[2] Genome assemblies of the isolates marked with an * were used for the identification of centromeric repeats *CentA* and *CentB* by BLAST searches.

[3] Sequencing reads of the isolates marked with an * were mapped against the Bgt_genome_v3_16 for coverage analyses.

Table S5 Presence of centromeric tandem repeats *CentA* and *CentB* in various *Blumeria graminis formae speciales.*

Forma specialis	**Number of isolates[1]**	**CentA[2]**	**CentB[2]**
B.g. tritici	13	91 - 97	87 - 97
B.g. tritici2 (dicocci)	6	90 - 95	87 - 92
B.g. triticale	5	87 - 95	89 - 93
B.g. dactylidis	1	93	89
B.g. secalis	5	89 - 93	0
B.g. hordei	1	0	0
B.g. avenae	1	0	0
B.g. lolii	1	0	0
B.g. poae	1	0	0

[1] Number of isolates of the respective *forma specialis* used as database for blast searches.
[2] Percentage of sequence identity obtained by blastn searches of *CentA* and *CentB* against *de novo* assembly of the respective *formae speciales*.

Table S6 Numbers of homologs of candidate effector genes and non-effector genes in the two Leotiomycetes *Botrytis cinerea* and *Phialocephala subalpina*

Gene group	Genes[a]	*B. cinerea*[b]	*P. subalpina*[c]	Common[d]	EffectorP[e]
Group 1 effectors	412	21 (5.1%)	18 (4.4%)	18	319 (77.4%)
Group 2 effectors	243	9 (3.7%)	10 (4.1%)	9	21 (8.6%)
Weak effectors	460	2 (0.4%)	2 (0.4%)	1	221 (48.0%)
Non-effector genes	7164	5200 (72.6%)	5571 (77.8%)	5136	N.A.
Non-effector genes with SP[f]	213	181 (85.0%)	182 (85.4%)	178	30 (14.1%)

[a]Number of genes in group.

[b]Number of *B. cinerea* homologs at the protein level using a blast E-value cutoff of 10E-6.

[c]Number of *P. subalpina* homologs at the protein level using a blast E-value cutoff of 10E-6.

[d]Number of genes that occur in the homolog sets of both *B. cinerea* and *P. subalpina.*

[e]Number and percentage of sequences that are predicted to be effectors by effectorP. (N.A. = not applicable)

[f]Non-effector genes that encode a protein with a predicted signal peptide.

Table S7 Summary of the genetic information for the 11 chromosomes of *B.g. tritici* from the cross 96224 X THUN-12.

Chromosome	Size[1]	cM	cM/Mb[2]	Number of SNPs	Marker density[3]	Centromere position	Centromere size[4]
Bgt_chr-01	12.0	405.3	33.7	9,552	1,257.4	4,615,073 – 6,421,201	1.8
Bgt_chr-02	15.6	519.4	33.3	14,648	1,064.1	3,069,147 – 4,106,259	1.0
Bgt_chr-03	5.9	223.7	37.7	5,838	1,016.0	4,259,975 – 5,764,197	1.5
Bgt_chr-04	11.1	332.9	30.1	11,654	950.1	3,843,054 – 5,051,318	1.2
Bgt_chr-05	19.5	547.0	28.1	16,373	1,190.2	10,692,477 – 12,182,665	1.5
Bgt_chr-06	11.6	404.4	34.8	8,085	1,437.9	5,089,060 – 6,816,649	1.7
Bgt_chr-07	16.2	507.2	31.3	12,477	1,300.8	4,089,458 – 6,155,798	2.1
Bgt_chr-08	11.9	366.4	30.8	12,290	968.5	10,057,310 – 10,798,263	0.7
Bgt_chr-09	19.7	503.2	25.5	14,200	1,388.9	7,758,199 – 10,180,824	2.4
Bgt_chr-10	10.2	302.6	29.5	11,260	911.1	927,128 – 2,336,503	1.4
Bgt_crh-11	3.6	167.4	46.8	2,646	1,351.3	1,859,683 – 2,542,942	0.7
Bgt_chr-Un	3.4	-	-	-	-	-	-

[1] Sizes of chromosomes are indicated in Mb.
[2] Recombination rate is indicated in cM/Mb.
[3] Marker density is indicated in SNP/Mb
[4] Centromere size is indicated in Mb

Table S8 Description of the recombination hotspots that were validated by PCR as described in Note S1.

Region Identifier	Chromosome	Genetic distance[1]	Physical distance[2]	Position SNP1	Position SNP2
RH1	Bgt_chr-04	5.9	280	2,448,054	2,448,334
RH2	Bgt_chr-10	5.0	286	781,096	781,382
RH3	Bgt_chr-10	5.0	341	785,348	785,689
RH4	Bgt_chr-06	5.0	496	7,904,937	7,905,433
RH5	Bgt_chr-06	5.9	531	564,855	565,386
RH6	Bgt_chr-10	5.0	1,097	781,793	782,890
RH7	Bgt_chr-09	6.0	1,290	989,596	990,886
RH8	Bgt_chr-02	7.6	1,313	5,082,461	5,083,774
RH9	Bgt_chr-01	5.1	1,567	6,463,488	6,465,055
RH10	Bgt_chr-05	6.8	1,637	19,279,245	19,280,882
RH11	Bgt_chr-07	6.9	1,746	9,580,758	9,582,504
RH12	Bgt_chr-01	8.5	1,785	4,124,946	4,126,731
RH13	Bgt_chr-07	6.1	1,987	6,296,885	6,298,872
RH14	Bgt_chr-09	5.2	2,164	19,051,181	19,053,345
RH15	Bgt_chr-04	6.8	2,170	3,182,062	3,184,232
RH16	Bgt_chr-06	7.6	2,193	4,982,554	4,984,747
RH17	Bgt_chr-05	7.7	2,195	946,490	948,685
RH18	Bgt_chr-08	6.1	2,201	3,110,760	3,112,961
RH19	Bgt_chr-10	7.7	2,397	764,237	766,634

[1] Genetic distance between SNP1 and SNP2 in cM

[2] Physical distance between SNP1 and SNP2 in bp.

Table S9 Primers used to verify the recombination hotspots by PCR.

	Primer for amplification[2]		Additional sequencing primers[3]	
Region[1]	Fwd primer	Rev primer	Fwd primer	Rev primer
RH1	CCATTAAAACATCACGCTACG	CCTTCGTCCCTTCATCC		
RH2	GGTGTGATTAGGGCATGC	GTTCCTAACGGCATTGTAGTG		
RH3	GAAACAAGCCATCCAGAAGG	CAAGACATGAAATGGCAAGAG		
RH4	CATACGAACTTGTCTAGTGCTGC	CACGATACATTTACATTACGAA		
RH5	CGATACATTTCCTCTCCATGG	CCACTCTCTAATTGGTCGAGAG		
RH6	ACTACAATGCCGTTAGGAACAG	CCAATCTACAAGATTGAGGTGT	CATGGAGTATCATTGGATA	
RH7	CGGATTTATAGCTATGATTGTTCG	AACGAGCCTCCAGTTGAG	CATCAAGGATCTTAGACTT	GCTTCATGATAATGGTGATAG
RH8	CAAAATTGAAGAAAGGAATACGTA	CACACACTAGTATCAACCCTAG	CGAAACTTTGCATTGAAGA	
RH9	GGAAGCTTGAAGATCGCAG	ACAAATCTCAAGCACTTGAGC		
RH10				
RH11	GAAAGTTTCCAATTAGGATCGTC	CGAAATCTGCGTGTCTGG		GAGTCTATGACTATCTAGCAC
RH12	CAAACATGTGAACTGACACAGG	CGATACGATCTTGTGTGTACG		
RH13	ATCAAGTGTTGCCTTACAAATAGG	GCAGAAAGGATTTCTGAGCAG		
RH14	GTAAGGAGACTCATGAGTCATGC	CGACGTGCTCGCTAAGG		
RH15	AAGCACGGAATCTTACCTATGTC	GCTACACGTCTATGTGGACC	GTACTCGTGATCTCGTAGA	GGACTCGCTTGCTTACACC
RH16	TCATACGAGACTTACTGCATCG	GGACATTCCGTAACTGTCG		AGCAAGAATGACTCAGTATA
RH17	GGCTTGGAGTGCAAGTCA	CAGTCCCTGAAGCTTAACATCA		
RH18	TGATTGCTCACATGGTGTTG	TGGTTGGATAGAGCAATTCC	GCTGAGATAGAGAATCAA	CTGCACTCTCGCAAGC
RH19	GGACAATCAATCAACCAGACC	GATTCATGGTGCGTTTATTCTG		

Table S10 Recombination frequency in the 96224 X THUN-12 depending of the genomic origin of THUN-12. THUN-12 is a hybrid between *B.g. tritici* and *B.g. secalis genotypes.*

Genomic origin[1]	Number of windows[2]	cM/50kb[3]
Mixed	34	4.55
B.g. secalis	540	1.05
B.g. tritici	2180	1.51

[1] Genomic origin of the isolate THUN-12, Mixed designate windows in which genotypes originate from *B.g. secalis* changes to *B.g. tritici* or vice-versa

[2] Number of 50kb windows

[3] Average recombination frequency in the 96224 X THUN-12 population across the windows

Table S11 Conservation of recombination hotspots in three mapping populations. Regions displaying high recombination rate (>3cM/50kb) in the 96224 X 94202 or 96224 X JIW2 mapping populations were compared to the corresponding regions in 96224 X THUN-12.

96224 X 94202[1]							96224 X THUN-12					
Linkage group[2]	Marker 1[3]	Marker 2[3]	Distance[4]	cM[5]	cM/50kb[6]		Chromosome[7]	Marker 1[8]	Marker 2[8]	Distance[4]	cM[5]	cM/50kb[6]
LG13	M226RE	M226LE	105003	19.8	9.4		Bgt_chr-08	snp94251	snp94380	111921	20.2	9.0
LG11	M132LE	M132RE	57603	3.6	3.1		Bgt_chr-09	snp109041	snp109109	59449	11.1	9.3
LG10	M168LE	M168RE	50509	5.6	5.6		Bgt_chr-08	snp90895	snp90951	50509	1.0	1.0
LG4	M273LE	M324MI	78891	7.3	4.6		Bgt_chr-10	snp111354	snp111485	78867	7.6	4.8
LG2	M205LE	M306RE	230564	14.9	3.2		Bgt_chr-02	snp11796	snp12078	227492	29.8	6.6
LG1	M056LE	M056RE	48988	3.2	3.3		Bgt_chr-06	snp65362	snp65404	52116	5.5	5.3
LG1	M219RE	M219LE	221178	13.6	3.1		Bgt_chr-06	snp64235	snp64487	222783	35.8	8.0

96224 X JIW2[9]							96224 X THUN-12					
Linkage group[2]	Marker 1[3]	Marker 2[3]	Linkage group[2]	Marker 1[3]	Marker 2[3]	Linkage group[2]	Marker 1[3]	Marker 2[3]	Linkage group[2]	Marker 1[3]	Marker 2[3]	cM/50kb[6]
LG2	M226LE	M226RE	105003	21.1	10.0		Bgt_chr-08	snp94251	snp94380	111921	20.2	9.0
LG2	M177RE	M636LE	174776	11.0	3.1		Bgt_chr-08	snp87522	snp87865	174394	18.3	5.3
LG4	M132RE	M132LE	57603	4.1	3.5		Bgt_chr-09	snp109041	snp109107	57687	11.1	9.6
LG6	M205LE	M306RE	230564	20.9	4.5		Bgt_chr-02	snp11793	snp12078	227951	29.8	6.5
LG7	M173LE	M173RE	29588	3.2	5.4		Bgt_chr-05	snp54670	snp54717	30731	5.4	8.7
LG7	M355RE	M355LE	37531	4.1	5.5		Bgt_chr-05	snp51635	snp51579	37670	7.9	10.5

[1] Conservation of high recombining regions (>3cM/50kb) in the 96224 X 94202 and 96224 X THUN-12 populations is higher than expected by chance (χ-square goodness-of-fit test, p=1.481e-06)
[2] indicates linkage group of the genetic maps described in Bourras et al., 2015
[3] Names of the genetic marker described in Bourras *et al.*, 2015
[4] Physical distance between the markers
[5] Genetic distance between the markers
[6] Estimated recombination frequency between the markers
[7] Chromosome that corresponds to the physical location of the marker in [3] in the 96224 assembly
[8] Names of the genetic markers in 96224 X THUN-12 corresponding to the marker in [3]
[9] Conservation of high recombining regions (>3cM/50kb) in the 96224 X JIW2 and 96224 X THUN-12 populations is higher than e xpected by chance (χ-square goodness-of-fit, p= 3.674e-07)

Table S12 Conservation of recombination coldspots in three mapping populations. Regions with no recombination (=0 cM) in the 96224 X 94202 or 96224 X JIW2 mapping populations were compared to the corresponding regions in 96224 X THUN-12.

96224 X 94202[1]						96224 X THUN-12					
Linkage group[2]	Marker 1[3]	Marker 2[3]	Distance[4]	cM[5]	cM/50kb[6]	Chromosome[7]	Marker 1[8]	Marker 2[8]	Distance[4]	cM[5]	cM/50kb[6]
LG1	M108LE	M037LE	125364	0.0	0.0	Bgt_chr-06	snp60911	snp60952	127627	2.002	0.8
LG2	M351RE	M090LE	144383	0.0	0.0	Bgt_chr-02	snp17481	snp17760	145394	0.0	0.0
LG2	M099LE	M099RE	167931	0.0	0.0	Bgt_chr-02	snp13020	snp13186	168066	0.0	0.0
LG2	M068LE	M276LE	38469	0.0	0.0	Bgt_chr-02	snp11439	snp11466	42088	0.0	0.0
LG3	M003LE	M181RE	556957	0.0	0.0	Bgt_chr-09	snp103361	snp103802	563128	0.848	0.1
LG3	M276RE	M263MI	132690	0.0	0.0	Bgt_chr-09	snp101500	snp101610	133409	0.0	0.0
LG4	M331RE	M331LE	230836	0.0	0.0	Bgt_chr-10	snp113551	snp113784	234342	3.3	0.7
LG7	M057RE	M088LE	398959	0.0	0.0	Bgt_chr-04	snp41685	snp42154	399501	18.8	2.3
LG10	M179LE	M636RE	254000	0.0	0.0	Bgt_chr-08	snp86860	snp87306	254517	2.2	0.4
LG10	M444MI	M140LE	199856	0.0	0.0	Bgt_chr-08	snp83594	snp83992	194336	0.0	0.0
LG11	M174LE	M283LE	274016	0.0	0.0	Bgt_chr-09	snp107692	snp107838	283704	1.7	0.3
LG14	M189RE	M189LE	120937	0.0	0.0	Bgt_chr-07	snp71103	snp71206	121934	0.0	0.0
LG15	M175RE	M175LE	52599	0.0	0.0	Bgt_chr-04	-	-	-	-	-
LG15	M143LE	M143RE	25629	0.0	0.0	Bgt_chr-04	snp33292	snp33338	28454	0.0	0.0

96224 X JIW2[9]						96224 X THUN-12					
Linkage group[2]	Marker 1[3]	Marker 2[3]	Distance[4]	cM[5]	cM/50kb[6]	Chromosome[7]	Marker 1[8]	Marker 2[8]	Distance[4]	cM[5]	cM/50kb[6]
LG1	M331RE	M781RE	177379	0.0	0.0	Bgt_chr-10	snp113551	snp113379	181132	0.8	0.2
LG1	M045LE	M464MI	326882.0	0.0	0.0	Bgt_chr-06	snp66663	snp66849	328023	0.8	0.1
LG1	M660LE	M155RE	171254	0.0	0.0	Bgt_chr-06	snp65495	snp65640	171251	0.0	0.0
LG2	M636RE	M179RE	200842	0.0	0.0	Bgt_chr-08	snp86950	snp87331	212600	0.0	0.0
LG3	M087RE	M707MI	66225	0.0	0.0	Bgt_chr-09	snp96837	snp96915	71202	0.9	0.6
LG3	M181LE	M181RE	194476	0.0	0.0	Bgt_chr-09	snp101608	snp101680	70585	0.0	0.0
LG3	M174RE	M283LE	354985	0.0	0.0	Bgt_chr-09	snp103361	snp103455	203659	0.8	0.2
LG3	M181LE	M181RE	194476	0.0	0.0	Bgt_chr-09	snp103589	snp103801	195911	0	0.0
LG3	M174RE	M283LE	354985	0.0	0.0	Bgt_chr-09	snp107693	snp107857	360875	4.2	0.6
LG7	M091LE	M320LE	329006	0.0	0.0	Bgt_chr-05	snp50796	snp50925	331802	2.0	0.3
LG9	M189LE	M189RE	120937	0.0	0.0	Bgt-chr-07	snp71103	snp71206	121934	0.0	0.0
LG10	M011RE	M057LE	155936	0.0	0.0	Bgt_chr-02	snp21256	snp21404	154554	0.0	0.0
LG11	M073RE	M323LE	247070	0.0	0.0	Bgt_chr-07	snp80248	snp80465	260878	0	0.0
LG11	M465LE	M465RE	61142	0.0	0.0	Bgt_chr-07	-	-	-	-	-
LG14	M012LE	M254LE	120188	0.0	0.0	Bgt_chr-01	snp1944	snp2053	120699	0.0	0.0
LG17	M351LE	M090LE	143241	0.0	0.0	Bgt_chr-02	snp17487	snp17760	144027	0.0	0.0

[1] Conservation of recombination coldspots (=0cM/50kb) in the 96224 X 94202 and 96224 X THUN-12 populations is higher than expected by chance (χ-square goodness-of-fit, p= 0.040)

[2] indicates linkage group of the genetic maps described in Bourras et al., 2015

[3] Names of the genetic marker described in Bourras et al., 2015

[4] Physical distance between the markers

[5] Genetic distance between the markers
[6] Estimated recombination frequency between the markers
[7] Chromosome that corresponds to the physical location of the marker in [3] in the 96224 assembly
[8] Names of the genetic marker in 96224 X THUN-12 corresponding to the marker in [3]
[9] Conservation of recombination coldspots (=0cM/50kb) in the 96224 X JIW2 and 96224 X THUN-12 populations is higher than expected by chance (**χ**-square goodness-of-fit, p=0.0022)

Table S13 Description of the 16 candidate effector gene families with at least 10 members in *B.g. tritici*.

Family	Number of *B.g. tritici* genes	Number of *B.g. hordei* genes	Total genes	Number of clusters	Mantel test simulated p-value	Group	Known members
E003	68	45	113	8	9.99E-05	Group-1	BEC1016, CSEP0254
E004	51	43	94	7	9.99E-05	Group-1	BEC1038
E008	40	13	53	8	0.019	Group-1	$AvrPm3^{a2/f2}$
E011	32	9	41	2	9.99E-05	Group-1	-
E014	17	15	32	4	9.99E-05	Group-1	AvrPm2, Avra13, BEC1011, BEC1054
E012	15	21	36	3	9.99E-05	Group-1	-
E020	13	7	20	1	0.868	Group-1	$SvrPm3^{a1/f1}$
E018	11	11	22	3	0.02	Group-1	$AvrPm3^{b2/c2}$
E036	10	2	12	1	0.117	Group-1	-
E001	105	99	204	18	9.99E-05	Group-2	-
E005	27	56	83	2	9.99E-05	Group-2	-
E007	27	26	53	6	0.001	Group-2	-
E015	22	9	31	2	0.004	Group-2	-
E013	20	13	33	4	9.99E-05	Group-2	-
E026	10	4	14	1	0.144	Group-2	-
E016	26	0	26	6	0.957	-	-

Table S14 Summary of duplicated genes in the reference assembly Bgt_genome.v3.16

Gene Class	Genome-wide	Percentage of identity		
		100	≥98	≥95
Non-effector	7165	155[1] (<2.2e-16)	437[1] (<2.2e-16)	679[1] (<2.2e-16)
Group 1	412	28[2] (0.0005)	81[2] (3.4e-12)	100[2] (3.8e-10)
Group 2	243	3	15	16[2] (0.007)
Other effector	189	7	11	16
Weak effector	460	97[2] (<2.2e-16)	170[2] (<2.2e-16)	239[2] (<2.2e-16)

[1] indicates that this group of genes is significantly reduced among the duplicated genes (p<0.05, exact binomial test), p-values are indicated in brackets

[2] indicates that this group of genes is significantly enriched among the duplicated genes (p<0.05, exact binomial test), p-values are indicated in brackets

Table S15 Copy number variation of genes in 36 isolates of *B.g. tritici*.

Gene Class	Genome-wide	Deletion	Duplication
Non-effector	7165	186[1]	298[1]
Group 1	412	38[2]	93[2]
Group 2	243	18	18
Other effector	189	5	6
Weak effector	460	50[2]	84[2]

[1] indicates that this group of genes is significantly reduced among the duplicated/deleted genes ($p<0.05$, exact binomial test)

[2] indicates that this group of genes is significantly enriched among the duplicated/deleted genes ($p<0.05$, exact binomial test)

Table S16 Estimates of nucleotide polymorphism rates in synonymous sites of genes derived from sequences of 36 *B.g. tritici* isolates.

All genes

	Genes[a]	Tot. len.[b]	Syn. Sites[c]	Syn. Mut[d]	Sites/kb[e]	Mut./Site[f]	Mut./kb[g]
Non-effector genes	6272	8412428	424090	19239	50.4	0.0151	0.2286
Group 1	345	141451	6542	340	46.2	0.0173	0.2403
Group 2	232	228144	10027	463	44.0	0.0154	0.2029
Group 1 expected[h]	n.a	n.a	7131	297	n.a	n.a	n.a
Group 2 expected[i]	n.a	n.a	11501	455	n.a	n.a	n.a
P-value exp. vs. obs. 1	n.a	n.a	< 0.00001	0.0858	n.a	n.a	n.a
P-value exp. vs. obs. 2	n.a	n.a	< 0.00001	0.7900	n.a	n.a	n.a

Single-copy genes

	Genes[a]	Tot. len.[b]	Syn. Sites[c]	Syn. Mut[d]	Sites/kb[e]	Mut./Site[f]	Mut./kb[g]
Non-effector genes	6048	8207325	414278	18746	50.5	0.0151	0.2284
Group 1	266	110707	5128	288	46.3	0.0180	0.2601
Group 2	210	206997	9083	407	43.9	0.0149	0.1966
Group 1 expected[h]	n.a	n.a	5588	232	n.a	n.a	n.a
Group 2 expected[i]	n.a	n.a	10449	411	n.a	n.a	n.a
P-value exp. vs. obs. 1	n.a	n.a	< 0.00001	0.0132	n.a	n.a	n.a
P-value exp. vs. obs. 2	n.a	n.a	< 0.00001	0.8879	n.a	n.a	n.a

[a]Total number of genes used for the analysis. (n.a. = not applicable)

[b]Total length of aligned sequences. (n.a. = not applicable)

[c]Number of synonymous sites in aligned sequences.

[d]Number of nucleotide polymorphisms in synonymous sites. (n.a. = not applicable)

[e]Number of synonymous sites per kb of aligned sequences. (n.a. = not applicable)

[f]Number of Nucleotide polymorphisms per number of synonymous sites. (n.a. = not applicable)

[g]Number of Nucleotide polymorphisms per kb of aligned sequences. (n.a. = not applicable)

[h]Value expected for group 1 candidate effector genes based on the values for non-effector genes.

[i]Value expected for group 1 candidate effector genes based on the values for non-effector genes.

Table S17 Nucleotide polymorphism rates in *B.g. tritici* genes from 36 isolates. Group 1 and 2 candidate effector genes, as well as all non-effector genes were treated as separate groups.

	**Non-effectors**	**Group 1**	**Group 2**
Genes[a]	6272	301	232
N[b]	43524373	443156	1154690
S[c]	21832364	216334	557761
S_d[d]	35506	626	982
N_d[e]	60368	2893	3546
p_N[f]	0.00139	0.00653	0.00307
p_S[g]	0.00163	0.00289	0.00176
p_N/p_S[h]	0.852	2.26	1.74

[a]Number of genes that showed polymorphisms in 36 *B.g. tritici* isolates.
[b]Number of non-synonymous sites in alignments between polymorphic genes and reference sequence.
[c]Number of synonymous sites in alignments between polymorphic genes and reference sequence.
[d]Number of non-synonymous differences between polymorphic genes and reference sequence.
[e]Number of synonymous differences between polymorphic genes and reference sequence.
[f]Proportion of non-synonymous differences per non-synonymous sites.
[g]Proportion of synonymous differences per synonymous sites.
[h]Ratio of non-synonymous to synonymous differences.

Table S18 Nucleotide polymorphism rates in *B.g. tritici* genes from 36 isolates. Families of candidate effector genes were treated as separate groups. The column “Genes” indicates the number of genes in a family that show polymorphisms in the 36 isolates. Definitions of the other values are the same as in Table S17.

Query	Group	Genes	N	S	Nd	Sd	pN	pS	pN/pS
E003	1	46	38577	18990	185	69	0.00479	0.00363	1.319
E004	1	38	52367	24717	312	70	0.00595	0.00283	2.103
E008	1	20	26793	13085	228	41	0.00850	0.00313	2.715
E011	1	20	30700	14881	123	50	0.00400	0.00335	1.192
E012	1	12	14080	6787	52	28	0.00369	0.00412	0.895
E014	1	10	10519	5254	56	30	0.00532	0.00570	0.932
E018	1	7	8713	4189	82	11	0.00941	0.00262	3.583
E020	1	11	24046	11863	145	44	0.00603	0.00370	1.625
E021	1	5	5700	2897	26	19	0.00456	0.00655	0.695
E022	1	3	6519	3098	39	9	0.00598	0.00290	2.059
E024	1	6	16222	8056	60	33	0.00369	0.00409	0.902
E025	1	4	1719	836	8	1	0.00465	0.00119	3.892
E028	1	5	13086	6554	70	16	0.00534	0.00244	2.191
E029	1	4	4627	2273	22	6	0.00475	0.00263	1.801
E030	1	5	9868	4739	82	18	0.00830	0.00379	2.187
E032	1	6	8805	4301	44	11	0.00499	0.00255	1.954
E033	1	7	5281	2527	25	7	0.00473	0.00276	1.709
E034	1	4	2723	1359	12	5	0.00440	0.00367	1.198
E039	1	2	3677	1761	21	5	0.00570	0.00283	2.011
E036	1	5	3532	1804	19	2	0.00537	0.00110	4.852
E038	1	6	10362	4907	25	6	0.00241	0.00122	1.973
E039	1	2	3681	1757	21	5	0.00570	0.00284	2.005
E041	1	3	5631	3029	6	7	0.00106	0.00231	0.46
E042	1	6	7388	3516	40	14	0.00541	0.00398	1.359
E045	1	6	9251	4558	58	5	0.00626	0.00109	5.715
E046	1	5	6571	3163	20	5	0.00304	0.00158	1.925
E047	1	1	1223	564	5	6	0.00408	0.01063	0.384
E048	1	1	605	312	2	0	0.00330	0.00000	n.a.[1]
E050	1	3	3391	1573	19	7	0.00560	0.00444	1.258
E053	1	5	9022	4771	23	45	0.00254	0.00943	0.27
E066	1	3	4322	2064	11	2	0.00254	0.00096	2.626
E001	2	85	349021	168862	866	341	0.00248	0.00201	1.228
E005	2	24	134691	64307	402	81	0.00298	0.00125	2.369
E007	2	27	149724	72968	357	112	0.00238	0.00153	1.553
E013	2	20	117285	55187	310	83	0.00264	0.00150	1.757
E015	2	18	75756	36494	220	57	0.00290	0.00156	1.859
E019	2	6	35927	18351	131	54	0.00364	0.00294	1.239
E026	2	8	32159	15555	52	10	0.00161	0.00064	2.515
E027	2	8	51209	25287	122	31	0.00238	0.00122	1.943
E031	2	5	28647	14627	119	17	0.00415	0.00116	3.574
E040	2	5	19868	9590	77	26	0.00387	0.00271	1.429
E061	2	4	15538	7267	31	39	0.00199	0.00536	0.371

[1] n.a. = not applicable

Table S19 Enrichment of candidate effector among differentially expressed genes.

	96224 vs 94202	JIW2 vs 94202	96224 vs JIW2	Total
**All genes**	161	180	197	339
**Non-Effector**	104[a]	114[a]	123[a]	220[a]
**Effector**	44[b]	49[b]	55[b]	87[b]
**Group 1**	29[b]	41[b]	39[b]	65[b]
**Group 2**	10[b]	6	12[b]	16[b]
**Other effectors**	5	2	4	6
**Weak effectors**	13	17[b]	19	32[b]

[a] indicates that this category of genes is significantly reduced in the differentially expressed genes compared to genome-wide level ($p<0.05$ for exact binomial test).

[b] indicates that this category of genes is significantly enriched in the differentially expressed genes compared to genome-wide level ($p<0.05$ for exact binomial test).

Table S20 Enriched TE superfamilies in up- and downstream regions of group 1 and 2 candidate effector genes as well as non-effector genes. P-values of chi2-test for enriched elements are given in parentheses.

Upstream

TE	Non-eff	Group 1	Group 2
RLC	39.97	76.41 (0.000517)	42.73
RLG	36.14	71.88 (0.000445)	43.05
RSX	182.19	269.47 (<0.00001)	364.14 (<0.00001)

Downstream

TE	Non-eff	Group 1	Group 2
RLC	48.48	162.48 (<0.00001)	58.71
RLG	44.15	121.44 (<0.00001)	56.23
RII	76.29	149.1 (<0.00001)	151.48 (<0.00001)
RIJ	12.09	28.97 (0.007615)	27.11 (0.015791)
RSX	163.73	288.03 (<0.00001)	378.53 (<0.00001)

Table S21 Number of copy number variants (CNV) and templates for unequal crossing over (UECO). Templates for UECO are defined as copies of the same TE families in the same transcriptional orientation that are found up- and downstream of a gene. Such direct repeats can serve as templates for UECO and thus lead to duplications of single-copy genes.

Group	genes	CNV	UECO	CVN+UECO
Group 1 effectors	412	150 (36%)	182 (44%)	73 (49%)
Group 2 effectors	245	46 (19%)	114 (47%)	20 (43%)
Non-effectors	7171	547 (7.6%)	2333 (33%)	250 (46%)
P-value (1 vs. 3)		<0.0001	<0.0001	n.a.[1]
P-value (2 vs. 3)		0.00032	<0.0001	n.a.[1]

[1] n.a. = not applicable

Table S22 Expression levels of candidate effector and non-effector genes located near recombination breakpoints.

	Number of genes[2]					Candidate effector genes		Non-effector genes	
**Region[1]**	**Candidate effector**	**Group 1**	**Group 2**	**Other effector**	**Non-effector**	**Mean expr.[3]**	**Med. Expr.[4]**	**Mean expr.[3]**	**Med. Expr.[4]**
Genome-wide	844	412	243	189	7626	373.2	25.9	136.5	36.4
1kb	67[5]	35[5]	21[5]	11[5]	195[6]	363.1	37.8	132.6	15.4
2.5kb	159[5]	69[5]	53[5]	37[5]	499[6]	362.6	31.7	140.9	24.1
5kb	244[5]	107[5]	82[5]	55[5]	1057[6]	350.2	29.1	131.3	30
10kb	357	165[5]	114[5]	78[5]	1949[6]	344.8	28	133.2	31.9
50kb	651	313[5]	196[5]	142	5047[6]	351	27.4	135.5	36

[1] The different regions around recombination points are described in Supplementary Note E.
[2] Number of genes annotated in the respective regions.
[3] Mean expression in rpkm of three biological replicates of the *B.g. tritici* isolate 96224 at 2 days after infection.
[4] Median expression in rpkm of three biological replicates of the *B.g. tritici* isolate 96224 at 2 days after infection.
[5] indicates that this category of genes is significantly enriched in the flanking region of recombination breakpoints compared to genome-wide level ($p<0.05$ for exact binomial test).
[6] indicates that this category of genes is significantly reduced in the flanking region of recombination breakpoints compared to genome-wide level ($p<0.05$ for exact binomial test).

Method S1 Construction of the genetic map.

Two versions of the genetic map were created during the course of the work. The first genetic map served as backbone for the anchoring of the polished raw assembly contigs into chromosomes (v1). The second genetic map was used to study recombination rates in *Blumeria graminis* and was based on the mapping to the final genome assembly (v3.16) and corrected for possible genotyping errors (described below) (v2). Both maps were created using the filtered SNP markers (see Methods) using the program MSTmap (Wu *et al.*, 2008). The genetic map was estimated using the Kosambi's distance function (Wu *et al.*, 2008). The significance threshold for markers to be placed in one linkage group was set to p <10-6. Pairs or single markers that were grouped more than 15 cM away from any other linkage group were placed in a separate linkage group. These single marker groups were removed from our analysis. Estimation of missing data by the program was turned off. The genetic map used for anchoring the contigs was 4,910 cM in size. In high-density genetic maps, genotyping errors can lead to an inflation of the genetic map due to overestimation of recombination events. We therefore eliminated SNP markers with possible genotyping errors from our dataset. We removed single SNP markers when the genetic order of the marker did not correspond to the physical order of the chromosome (3% of the markers). If co-segregating markers groups with more than 10 SNP markers did not correspond to the physical orientation of the chromosome, they were retained in the genetic map. After removing of potential erroneous SNP markers, genetic distances were re-estimated using MSTmap for each linkage group separately. To test for potential gene conversion events, we detected potential double-crossovers that are less than 1 kb in size in the 96224 X THUN-12 genetic map. These intervals were tested for overlap with genes with the *Bedtools* intersect command (Quinlan & Hall, 2010)

Method S2 Transcriptome analysis.

We used published RNA-Seq data from three different *B.g. tritici* isolates (each with three biological replicates) after infection of the susceptible wheat variety Chinese Spring and harvested at 48 hours after infection (Praz *et al.*, 2018). Reads were mapped to the genome using STAR with the parameters: --outFilterMultimapNmax 10 --outFilterMismatchNoverLmax 0.02 --

alignIntronMax 500 (Dobin *et al.*, 2013). Read counts for all genes were obtained with featureCounts using standard parameters and the -M option allowing multi-mapping reads. Expression analyses were done using the R package edgeR as previously published (Praz *et al.*, 2018), and the same criteria were used for differential expression (log2FC > |1.5| and *p*-value (FDR) < 0.01).

Method S3 Phylogenetic analyses.

Protein sequence alignments were performed with Muscle 3.8.320. Phylogenetic trees were inferred with RaxML 8.2.8 using a protein GTR model and 100 bootstraps (Stamatakis, 2014). Trees were visualized using FigTree v1.4.1 (http://tree.bio.ed.ac.uk/software/figtree/) and phylogenetic clusters inside individual candidate effector families were identified manually. Pairwise physical and phylogenetic distances were calculated within the 21 largest candidate effector gene families. If two genes are located on the same chromosome, the physical distance between them was calculated using the positions in the middle of the genes, if two genes are located on different chromosomes, the physical distance between them was arbitrary set to 1000000. The phylogenetic distance between two genes was calculated based on the phylogenetic tree of the family as the sum of the lengths of the branches that separate the two genes.

Method S4 Statistical analyses.

The binomial test from the R base (Team, 2008) package was used for the analysis of enrichment of candidate effectors around recombination breakpoints and among duplicated and deleted genes. For the binomial test, the probability of success was calculated by calculating the proportion of each gene class contributing to the number of duplication and deleted genes respectively. Correlation between pairwise genetic and physical distances of members of candidate effector families was tested using Mantel test (R package ade4, (Dray & Dufour, 2007). Significance of differences in polymorphism rates between candidate effector and non-effector genes was tested with a χ-square test. To test if recombination hotspot and coldspots are conserved between different mapping population, 100 random intervals of the size of the

intervals in Table S11 and S12 were simulated with the *Bedtools* random command (Quinlan & Hall, 2010)). The corresponding intervals in the 96224 X THUN-12 genetic map were identified with *Bedtools* interval command. χ-square goodness-of-fit test was used to test if observed conservation of hot and coldspots are higher than expected by chance. The proportion of recombination hotspots near centromere was also tested with a χ-square goodness-of-fit test.

Note S1 Verification of the integrity of the genome assembly in recombination hotspots by PCR.

In order to exclude artefacts from potential sequence mis-assemblies on the analysis of recombination rate we amplified a number of highly recombining regions by PCR and sequenced them. We selected 19 (RH1-19) marker pairs that were physically less than 2,500 bp apart from each other and had a genetic distance of more than 5cM. The selected genome regions are listed in Table S7. PCR primers for each region were designed to amplify PCR products which included both indicative SNP markers used for recombination rate analysis. Primers used for the amplification of highly recombinogenic genomic regions (RH 1-9 and 11-19) are listed in Table S8. PCR amplification was conducted on genomic DNA of the parental isolates 96224 and THUN-12 using Phusion High-Fidelity DNA Polymerase (New England Biolabs). PCR products were purified with the GenElute PCR Clean-up Kit (Sigma-Aldrich) and Sanger-sequenced on an ABI3730 DNA Analyser (Applied Biosystems) using Bright-Dye Terminator Mix (Nimagen) with the primers designed for initial PCR amplification. In a few cases additional sequencing primers was necessary to cover the indicative SNPs defined for recombination analysis (Table S8). Due to the highly repetitive nature of the target regions, we ensured primer specificity with blast against the assembly (v3.16). Regions that could not be sequenced with desired accuracy due to unspecific by-products were subcloned using the StrataClone Blunt PCR Cloning Kit (Agilent) before sequencing with either internal or vector primers (Table S8).

Except for one region (RH10) primers within the vicinity (100 – 1000bp) of the informative marker could be designed. For 17 out of the 18 remaining regions PCR amplification from both parental isolates was successful and resulted in an amplicon of the expected size (Fig. S8). The obtained PCR products were sequenced. We confirmed sequence polymorphisms at the marker positions through manual inspection of the obtained sequences. Thus, PCR amplification confirmed the sequence of our reference assembly and excluded the possibility that the recombination hotspots were caused by genome mis-assemblies.

Note S2 Analysis of sequence diversity of genes in 36 *B.g. tritici* isolates.

To sample diversity and polymorphism rates in candidate effector and non-effector genes, we mapped genomic sequences from the 36 previously published *B.g. tritici* isolates (Table S3) to the genome assembly. In total we identified 36,358 SNPs in coding sequences (CDS) of genes, and only 89 codons with more than one polymorphism. This relatively low level of sequence diversity (3.8 SNPs per kb CDS) prevented a meaningful analysis of the diversity in single genes. Instead, the 412 group 2 candidate effector genes, 245 group 2 candidate effector genes, and 7,171 non-effector genes were analysed as separate groups (i.e. lengths of genes numbers of polymorphisms were added up for each group). Weak candidate effector genes (see Note S4) were excluded from this analysis because the majority of them are derived from high-copy TEs which show high levels of polymorphisms between isolates. Erroneous mapping for TE reads to the reference genome can thus lead to frequent false positive SNP calling. An in-house perl script was used to determine the type of mutation in CDS (synonymous, non-synonymous). Using the method of (Nei & Gojobori, 1986), we determined the proportions of non-synonymous differences per non-synonymous sites and synonymous differences per synonymous sites. Here, sequences from all 35 isolates were aligned to the gene sequence of the reference isolate 96224. Numbers of synonymous and non-synonymous sites were calculated for all sequences. Numbers of synonymous (pS) and non-synonymous differences (pN) then determined for all sequence pairs. Because polymorphism levels between isolates were very low, numbers of synonymous (pS) and non-synonymous differences (pN) were added up for all members of the gene group examined (group 1 and group 1 candidate effectors, non-effector genes or individual gene families). From this, the ratio of non-synonymous to synonymous differences per site (p_N/p_S) was calculated for each examined gene group. Both group 1 and 2 candidate effector genes show more than twice the p_N/p_S values of non-effector genes (Table S16). The high p_N/p_S values of group 1 and 2 candidate effector genes could indicate that many of these genes are under positive selection.

Analysis of individual families of candidate effector genes showed that high p_N/p_S values are relatively consistent across all families (Table S17). This excludes the possibility that high p_N/p_S

values of an entire group are simply the result of extreme diversity in a small number of genes. Only a few (mostly small) families are outliers with low or very high p_N/p_S values. These are likely statistical fluctuations due to small numbers of nucleotide differences in these gene families. Nevertheless, some large candidate effector genes also have very high values (e.g. E008). Here, sequence data from additional isolates will be needed to determine whether these particular gene families indeed have such high rates of non-synonymous polymorphisms.

The two-speed genome hypothesis states that candidate effector genes may have higher overall polymorphism rates than non-effector genes. In some fungi, mutations can be induced if a gene is close to a TE that is silenced through the RIP pathway (Dong *et al.*, 2015). However, *B.g. tritici* lacks RIP pathway genes (Wicker *et al.*, 2013). To test whether candidate effector genes have higher mutation rates, we compared frequencies of nucleotide polymorphisms in synonymous sites in candidate effectors and non-effector genes. Here, we only considered fourfold degenerate codons where the third base can be substituted-freely by any base without causing an amino acid change (e.g. Leucine is encoded by codons starting with CT while the third position can be any of the four bases), which are the codons starting with CT, GT, TC, CC, AC, GC, CG, and GG.

If candidate effector genes indeed had overall higher mutation rates, one would expect higher numbers of polymorphisms in these sites. We found that candidate effector genes (group 1 and 2) do not have significantly higher substitution rates in synonymous sites than non-effector genes (Table S15). Curiously, non-effector genes had overall significantly more synonymous sites than candidate effector genes (Table S15). This is can be explained by differences in amino-acid compositions, because predicted group 1 and 2 effector proteins are depleted in amino-acids that are encoded by multiple codons (e.g. Leucine, Arginine and Serine) compared to predicted non-effector proteins (Fig. S15).

Because the numbers of polymorphisms can be distorted if sequences from paralogs are compared (e.g. a given isolate contains a different copy of a gene), we repeated the analysis using

only genes which show no evidence for copy number variations (Table S15). This had only little effect on the results.

Note S3 A chromosome-scale assembly of the *B.g. tritici* genome.

To obtain a high quality *B.g. tritici* reference genome assembly consisting of chromosome-sized scaffolds, we combined three experimental approaches. First, the genome of *B.g. tritici* isolate 96224 was sequenced to approximately 85-fold coverage with PacBio technology obtaining an average read length of 18’000 bp. These sequences were assembled into 653 sequence scaffolds with a cumulative length of 140.6 Mb. The N50 was 847.9 kb meaning that 50% of the assembly was in scaffolds larger than 847.9 kb, while the N90 was 190.4 kb (Table 1). Because of the high error rate of PacBio technology, the sequence scaffolds were polished with two runs of Illumina sequences that provided approximately 40-fold coverage of the genome. Second, we used a BAC library consisting of 23,040 clones that had been fingerprinted and end-sequenced (Wicker *et al.*, 2013). The BAC fingerprinting data was used earlier to construct 201 BAC contigs which had served as backbone of an initial draft of the *B.g. tritici* genome (Wicker *et al.*, 2013). BAC-end sequencing resulted in 20,001 useable sequences. Third, we created a high density genetic map of *Blumeria graminis* using 118 F1 progeny of a cross between *B.g. tritici* reference isolate 96224 and *B.g. triticale* isolate THUN-12 (Fig. 2). All progeny were sequenced individually to approximately 16-fold coverage with Illumina technology. High-throughput genotyping allowed to identify 123,159 SNP markers segregating in the mapping population (see Methods and Method S1). The SNP markers were used as genetic markers to construct a genetic map consisting of 11 linkage groups ranging in length from 188.6 to 732 cM and with a cumulative length of 4,910 cM.

The BAC end sequences were mapped to the PacBio sequence contigs by blastn, resulting in 17,917 BAC ends that could unambiguously be mapped. Positional information of BAC ends on PacBio contigs was used to form larger scaffolds (i.e. in cases were one end of a BAC was located on one scaffold while the other end matched to a different scaffold). In addition, positional information was used to identify 47 mis-assemblies in PacBio sequence contigs. Positions of mis-assemblies were also narrowed down based on SNP marker information. Manual examination of

10 cases showed that mis-assemblies were often caused by highly similar copies of transposable elements which range in size from 6-8 kb.

Information from BAC end sequencing and genetic mapping complemented each other and helped arranging PacBio contigs into large scaffolds. For example, PacBio contigs could be oriented via BAC ends if their orientation could not be determined through genetic map data (in regions of low recombination or for short contigs). Additionally, PacBio contigs connected by BAC ends could be integrated into scaffolds in the absence of genetic marker data (in regions with few polymorphisms).

In total, 313 PacBio contigs were assembled into 11 pseudomolecules which corresponded to the 11 genetic linkage groups (Table S3). The 313 contigs had an N50 of 873 kb (N90 of 223.7 kb) and represent 97.6% of the total sequence (Table 1). The un-anchored contigs were assembled into chromosome unknown (Chr-Un). The 11 pseudomolecules (hereafter equated with chromosomes) range in size from 3.6 to 19.7 Mb (Table S1) and contain only 357 sequence gaps The final genome version that is publicly available and used in this study will hereafter be referred to as Bgt_genome_v3.16.

We used the curated genetic map with a size of 4,279.5 cM to verify the quality of the assembly after scaffolding (see Method S1 for details on curation). We analyzed co-linearity between the order of the genetic and the physical position of markers (Fig. 2). A total of 99.8% of the markers in the genetic map were collinear with physical order in the genome assembly. We detected only four small discrepancies between genetic and physical positions (discrepancies of size: 27kb, 350kb, 1.7kb, 19kb) on chromosomes Bgt_chr-06, 07, 08, 09 respectively (Fig 2.). These four discrepancies were all located within individual PacBio sequence contigs. Because we could not determine whether they represent mis-assemblies or artefacts of the genetic map, they were not modified. The genetic map contains 119,023 markers, the largest chromosome Bgt_chr-09 is covered by 14,200 markers and the smallest Bgt_chr-11 by 2,646 markers, respectively.

Note S4 Genome size estimation.

The chromosome-scale assembly generated in this study has a size of 140.6 Mb which is a 71% size increase compared to the previous genome version of the same isolate (Wicker *et al.*, 2013). In the previous study, genome size was estimated to be 180 Mb based on BAC fingerprint assemblies. The much shorter cumulative length of the sequence assembly was assumed to be due to collapsed repeats. To obtain an estimate of the amount of collapsed repeats, we mapped Illumina reads onto the chromosome assembly. Sequence coverage with Illumina reads showed a narrow distribution for most of the genome with a mean of 22 and a standard deviation of 5.9 (Fig. S4). Only approximately 0.5% of genomic 500 bp windows had a sequence coverage higher than 44 (i.e. twice the mean coverage). Thus, we decided to use 44-fold coverage as a cutoff for sequences to be examined further. We identified 136 regions with a total length of 2.24 Mb which have sequence coverage of 44 or more, some having nearly 20-fold the average coverage. Most of these correspond to short PacBio contigs that were integrated in Chr-Un (Fig. S4a), indicating that they were also problematic for the PacBio assembly. Nevertheless, some repeat arrays were assembled in longer PacBio scaffolds and could be integrated into the pseudomolecules (Fig. S4a).

Some of regions with highest sequence coverage (indicative of strongly collapsed repeats) correspond to tandem repeat arrays of 5S and 45S ribosomal DNA (rDNA). One cluster of 45S rDNA genes is located on chromosome 9 approximately at position 16.4 Mb (Fig. 4). It is comprised of tandemly repeated 7.3. kb units containing the genes for 18S, 5.8S and 28S ribosomal RNA. This cluster has a size of 134.9 kb, but additional 45S rDNA arrays were also found on Chr-Un. We assume that these all actually belong to the same cluster on chromosome 9 but were not integrated in the assembly due to their highly repetitive nature. The cumulative length of all 45S rDNA repeats is 474 kb. The 45S rDNA regions have an average Illumina sequence coverage of 282-fold, from which we extrapolate that these sequences represent approximately 5.8 Mb of collapsed repeats (Table S2, see Methods). Thus, we estimate that *B.g. tritici* contains almost 800 copies of the 7.3 kb 45S rDNA cluster.

On chromosome 5 (approximately at position 14 Mb), we identified a cluster of tandemly repeated 2.8 kb sequences containing the 5S rDNA gene. Again, additional copies were also found

on Chr-Un. Sequence coverage indicates that the 343 kb of 5S rDNA sequences actually represent 3.74 Mb of collapsed repeats, corresponding to approximately 1,300 5S rDNA genes (Table S2).

The genetic centromeres contain large arrays of tandem repeats (example in Fig. S5) for which Illumina sequence coverage indicates that they are strongly collapsed Fig. S4, Fig S5, Table S2). We identified two types of centromeric tandem repeats: CentA has a size of 189 bp and is found in the centromeres of chromosomes 1 and 8, while CentB has a size of 197 bp and is found in the centromere of chromosomes 9 and 11 (Fig. S4). The 64 kb of assembled CentA and 14 kb CentB repeats probably represent approximately 440 kb and 76 kb of collapsed repeats (Table S2). However, we can not exclude the possibility that other centromeres also contain CentA and/or CentB repeats, as many of them could not be integrated into the chromosome assemblies and instead were included in Chr-Un. Different chromosome-specific centromeric repeats in the same species were reported before (Plohl *et al.*, 2014) and could witness an ancient hybridization event that was postulated previously (Menardo *et al.*, 2017). We identfied *CentA* and *CentB* repeats in various *formae speciales* of cereal mildews (Table S4 and S5). Interestingly, *B.g. dactylidis* contains both types, while *B.g. secalis* only contains *CentA* repeats, which supports the previously proposed scenario that the *forma specialis B.g. secalis* diverged from *B.g. tritici* before *B.g. dactylidis (Menardo et al., 2017)*.

In addition, we identified a novel 4.6 kb tandem repeat (DRC) which contains no genes but contributes approximately 1.16 Mb to the genome. It is likely that most DRC repeats are arranged in a single cluster on chromosome 4 because there were no other full-length copies on any other chromosome (Table S2).

Surprisingly, an additional 1.03 Mb represent sequences with very high sequence coverage but without clear repeat structure. Several of these loci share some stretches of near-identical sequences, but they contain no obvious tandem repeats or TEs. What is common to most of them is a highly fluctuating CG content (Fig. S4c). We hypothesize that either these sequences indeed represent collapsed repeats, or their sequence composition gave them a positive bias during the sequencing procedure and thus resulted in a higher coverage. Assuming that these are also collapsed repeats, then the 1.03 Mb would represent approximately 14.18 Mb of actual sequence.

Only few collapsed repeats (totaling 143 kb) are derived from TE sequences, indicating that the TE fraction was assembled well with PacBio technology. Thus, if all these collapsed sequences would be added to the assembly size, the *B.g. tritici* genome has a size of approximately 166.6 Mb. This value is lower than the previous estimate of 180 Mb which was based on BAC fingerprinting and Roche/454 sequence coverage (Wicker *et al.*, 2013). Mapping of BAC end sequence pairs to the pseudomolecules showed that BAC clones are on average approximately 20% shorter than the size estimated based on BAC fingerprinting. Additionally, we found that approximately 14% (22 Mb) of the pseudomolecule sequences were not covered by BAC clones. Thus, if BAC fingerprinting over-estimated genome size by 20%, the actual length covered by the BACs was 144 Mb. Adding the approximately 22 Mb that were not covered by BACs to this number results in an estimate of 166 Mb for the size of the whole genome, very close to the 166.6 Mb estimated from the PacBio and Illumina sequence data.

Note S5 Transposable element annotation.

In total, we identified 166 different TE families in *B.g. tritici.* For 106 of the 166 identified TE families, consensus DNA sequences could be generated (i.e. at least three full-length elements of a family were identified, which were then used to derive a consensus sequence). The 166 TE families occupy approximately 55% of the genome. However, homology search at the protein level (using predicted TE proteins from different superfamilies) in the un-annotated sequence indicated the presence of probably hundreds of additional low-copy TE families. Based on these data, we estimate that approximately 85% of the *B.g. tritici* genome is derived from TE sequences.

The repetitive fraction of the *B.g. tritici* genome is dominated by non-LTR retrotransposons of the *SINE* (short interspersed nuclear element) and *LINE* (long interspersed nuclear element) order (Fig. S2). *SINEs* are represented mainly by 10 families which are present in hundreds to thousands of copies. For example, the most abundant *SINE* family (*RSX_Lie*) is present in approximately 3,500 copies, representing 1.7% of the genome (Fig. S2). *SINEs* are evenly distributed along chromosome arms but mostly absent from centromeres (Fig. 1). *LINE* elements are found along the entire chromosomes with an enrichment in centromeric regions (Fig. 1). Interestingly, this enrichment is due to 11 *LINE* families which are highly specific for centromeres but absent from chromosome arms (for details of centromeric repeat composition see Fig. 1, Fig. S5). The other 46 identified *LINE* families are found almost exclusively on chromosome arms.

LTR retrotransposons of the *Gypsy* superfamily are also mainly found on chromosome arms, showing a similar distribution as *SINEs*, while LTR retrotransposons of the *Copia* superfamily are found all across the genome with no obvious enrichment in any chromosomal compartment (not shown). While retrotransposons show an extreme diversity in the *B.g. tritici* genome, we only identified three families of Class II (DNA) transposons, all of them belonging to the *Mariner* superfamily. They contribute only approximately 1% to the total genome sequence. A more detailed analysis of the TE fraction of the *B.g. tritici* genome will be published elsewhere.

Note S6 Gene annotation and candidate effector classification.

To annotate the new genome assembly, we combined different annotation approaches. We first transferred the gene annotation published in (Praz *et al.*, 2018) and manually curated it to the *B.g. tritici* genome v3.16. In parallel we used the annotation pipeline Maker 2.31.8 (Cantarel *et al.*, 2008) to annotate genes in the genome using the protein databases of *B.g hordei* and *B.g. tritici* as templates (Spanu *et al.*, 2010; Wicker *et al.*, 2013). We used the *B. graminis* repeat database (integrated in TREP, botinst.uzh.ch/en/research/genetics/thomasWicker/trep-db.html) to mask repeats during the annotation. We then excluded all genes shorter than 50 bp and compared genes from the original annotation with genes annotated by Maker using a bi-directional blast search. All genes for which the reciprocal blast results were ambiguous were then manually curated using published RNA-Seq data from isolate 96224 (Praz *et al.*, 2018).

BUSCO v3.0.2 (Simao *et al.*, 2015) (accessed on July 2017) was used to assess the completeness of the genome assembly with the use of blastn (v2.2.31+), HMMER (v3.1b2, February 2015) and AUGUSTUS (v3.2.3). We used the following lineages: fungi_odb9, ascomycota_odb9 and pezizomycotina_odb9.

We focused especially on the identification and annotation of candidate effector genes as they play important roles in host-pathogen interactions (Jones & Dangl, 2006; Panstruga & Dodds, 2009; Giraldo & Valent, 2013). To identify so far undiscovered candidate effector genes in the new genome sequence, we performed an ab-initio search by screening the entire genome for open reading frames (ORFs) that encode an N-terminal signal peptide. This search resulted in the identification of 12,863 ORFs with signal peptides. These 12,863 ORFs were then filtered in the following steps: To filter out known genes, these ORFs were then used as queries in blastn searches against the already annotated genes. Then, only those were selected that were also supported by expression data (cutoff at 20 rpkm). Finally, these were then filtered for overlap with typical TE genes (i.e. genes that clearly corresponded to canonical TE genes were removed). Predicted ORFs that overlapped with annotated TEs were only retained if they fulfilled one or more of the following criteria: (i) the predicted ORF is in reverse orientation relative to the TE, (ii) the ORF is in the same orientation as the TE, but expression is only found in the region of the predicted ORF and not the rest of the TE, (iii) the predicted ORF only partially overlaps the

respective TE, or (iv) the ORF is in the same orientation as the TE but is encoded in a different reading frame than the canonical TE genes. This filtering of the initially identfied 12,863 ORFs with signal peptides resulted in the addition of 124 gene models to the genome annotation. The combination of the de novo annotation, transfer of the existing *B.g. tritici* annotation to the new genome and the annotation of ORFs with signal peptide resulted in the annotation of 8'470 genes.

Using the method described in (Praz *et al.*, 2017), all predicted *B.g. tritici* proteins were then used in blastp searches against the protein databases of *B.g hordei* (http://www.blugen.org/index.php?page=data, accessed 18.07.2017), *Podospora anserina* (http://podospora.igmors.u-psud.fr/download.php, accessed 18.07.2017) and *Neurospora crassa* (https://www.ncbi.nlm.nih.gov/genome/19, accessed 18.7.2017). After elimination of proteins with homology in the repeat database (Bg_repeats + PTREP16, e-value cut off e-05), we retained 6,166 proteins for *B.g. hordei*, 10,606 for *P. anserina* and 10,788 for *N. crassa.* We clustered the proteins in families following the pipeline described in (Praz *et al.*, 2017) and retained families with genes only from *B.g. tritici* and *B.g. hordei* as candidate effector genes.

The annotations of these candidate effector genes were manually curated, comparing the gene models with RNA-seq data, on the genome browser IGV14. The final annotation was then mapped to the genome with gmap to obtain a gff (general feature format) file.

After manual curation, we redefined candidate effector families using the *B.g. tritici* and *B.g. hordei* candidate effector genes using the same pipeline as before (with option -I 1.4). Families with only 10% or fewer member per family having a signal peptide were flagged as "weak" effector families as well as families with more than 50% of their members showing homology to repeats (based on CDS and protein blast searches against the databases nrTREP17 and PTREP17).

To study whether the identified candidate effector genes are conserved in more closely related fungal species, we searched for homologs in *Botrytis cinerea* (a necrotrophic fungus) and *Phialocephala subalpina* (a globally distributed root endophyte, (Schlegel *et al.*, 2016). Predicted protein sequences of Group 1 and 2, weak candidate effector genes and non-effector genes were used in blastp searches against the predicted proteins of *B. cinerea* and *P. subalpina* (accessed 09/19/2018 from ncbi.nlm.nih.gov/genome/), using an E-value cutoff of 10E-6. Despite the low

low stringency level, only 3.7%-5.1% of candidate effector genes have homologs in either of the two fungi. In contrast, 72.6% and 77.8% of the non-effector genes have homologs in the two fungi (Table S6).

As an independent assessment, we performed EffectorP (Sperschneider *et al.*, 2018) analysis on group 1 and 2, weak effectors and non-effector proteins with signal peptides (Table S6). In total, 77% of group 1 candidate effectors were predicted to be effectors, while only 21% of group 2 effectors were. Interestingly, also 48% of the weak effectors were predicted to be effectors. In contrast, only 14% of non-effector proteins with signal peptides were predicted to be effectors.

References

Bourras S, McNally KE, Ben-David R, Parlange F, Roffler S, Praz CR, Oberhaensli S, Menardo F, Stirnweis D, Frenkel Z, et al. 2015. Multiple Avirulence Loci and Allele-Specific Effector Recognition Control the *Pm3* Race-Specific Resistance of Wheat to Powdery Mildew. *Plant Cell* **27**(10): 2991-3012.

Cantarel BL, Korf I, Robb SMC, Parra G, Ross E, Moore B, Holt C, Alvarado AS, Yandell M. 2008. MAKER: An easy-to-use annotation pipeline designed for emerging model organism genomes. *Genome Research* **18**(1): 188-196.

Dobin A, Davis CA, Schlesinger F, Drenkow J, Zaleski C, Jha S, Batut P, Chaisson M, Gingeras TR. 2013. STAR: ultrafast universal RNA-seq aligner. *Bioinformatics* **29**(1): 15-21.

Dong SM, Raffaele S, Kamoun S. 2015. The two-speed genomes of filamentous pathogens: waltz with plants. *Current Opinion in Genetics & Development* **35**: 57-65.

Dray S, Dufour AB. 2007. The ade4 package: Implementing the duality diagram for ecologists. *Journal of Statistical Software* **22**(4): 1-20.

Giraldo MC, Valent B. 2013. Filamentous plant pathogen effectors in action. *Nature Reviews Microbiology* **11**(11): 800-814.

Jones JDG, Dangl JL. 2006. The plant immune system. *Nature* **444**(7117): 323-329.

Menardo F, Praz CR, Wyder S, Ben-David R, Bourras S, Matsumae H, McNally KE, Parlange F, Riba A, Roffler S, et al. 2016. Hybridization of powdery mildew strains gives rise to pathogens on novel agricultural crop species. *Nature Genetics* **48**(2): 201-205.

Menardo F, Wicker T, Keller B. 2017. Reconstructing the Evolutionary History of Powdery Mildew Lineages *(Blumeria graminis)* at Different Evolutionary Time Scales with NGS Data. *Genome Biology and Evolution* **9**(2): 446-456.

Nei M, Gojobori T. 1986. Simple methods for estimating the numbers of synonymous and nonsynonymous nucleotide substitutions. *Molecular Biology and Evolution* **3**(5): 418-426.

Panstruga R, Dodds PN. 2009. Terrific Protein Traffic: The Mystery of Effector Protein Delivery by Filamentous Plant Pathogens. *Science* **324**(5928): 748-750.

Plohl M, Mestrovic N, Mravinac B. 2014. Centromere identity from the DNA point of view. *Chromosoma* **123**(4): 313-325.

Praz CR, Bourras S, Zeng FS, Sanchez-Martin J, Menardo F, Xue MF, Yang LJ, Roffler S, Boni R, Herren G, et al. 2017. *AvrPm2* encodes an RNase-like avirulence effector which is conserved in the two different specialized forms of wheat and rye powdery mildew fungus. *New Phytologist* **213**(3): 1301-1314.

Praz CR, Menardo F, Robinson MD, Muller MC, Wicker T, Bourras S, Keller B. 2018. Non-parent of Origin Expression of Numerous Effector Genes Indicates a Role of Gene Regulation in Host Adaption of the Hybrid Triticale Powdery Mildew Pathogen. *Frontiers in Plant Science* **9**: 49.

Quinlan AR, Hall IM. 2010. BEDTools: a flexible suite of utilities for comparing genomic features. *Bioinformatics* **26**(6): 841-842.

Schlegel M, Munsterktter M, Guldener U, Bruggmann R, Duo A, Hainaut M, Henrissat B, Sieber CMK, Hoffmeister D, Grunig CR. 2016. Globally distributed root endophyte

Phialocephala subalpina links pathogenic and saprophytic lifestyles. *Bmc Genomics* **17**: 1015.

Simao FA, Waterhouse RM, Ioannidis P, Kriventseva EV, Zdobnov EM. 2015. BUSCO: assessing genome assembly and annotation completeness with single-copy orthologs. *Bioinformatics* **31**(19): 3210-3212.

Spanu PD, Abbott JC, Amselem J, Burgis TA, Soanes DM, Stuber K, van Themaat EVL, Brown JKM, Butcher SA, Gurr SJ, et al. 2010. Genome Expansion and Gene Loss in Powdery Mildew Fungi Reveal Tradeoffs in Extreme Parasitism. *Science* **330**(6010): 1543-1546.

Sperschneider J, Dodds PN, Gardiner DM, Singh KB, Taylor JM. 2018. Improved prediction of fungal effector proteins from secretomes with EffectorP 2.0. *Molecular Plant Pathology* **19**(9): 2094-2110.

Stamatakis A. 2014. RAxML version 8: a tool for phylogenetic analysis and post-analysis of large phylogenies. *Bioinformatics* **30**(9): 1312-1313.

Team RDC 2008. R: A language and environment for statistical computing.

Wicker T, Oberhaensli S, Parlange F, Buchmann JP, Shatalina M, Roffler S, Ben-David R, Dolezel J, Simkova H, Schulze-Lefert P, et al. 2013. The wheat powdery mildew genome shows the unique evolution of an obligate biotroph. *Nature Genetics* **45**(9): 1092 – 1096.

Wu YH, Bhat PR, Close TJ, Lonardi S. 2008. Efficient and Accurate Construction of Genetic Linkage Maps from the Minimum Spanning Tree of a Graph. *Plos Genetics* **4**(10): e1000212.
